# Identification of the Optimal Season and Spectral Regions for Shrub Cover Estimation in Grasslands

**DOI:** 10.3390/s21093098

**Published:** 2021-04-29

**Authors:** Irini Soubry, Xulin Guo

**Affiliations:** Department of Geography and Planning, University of Saskatchewan, 117 Science Place, Saskatoon, SK S7N 5C8, Canada; xug991@mail.usask.ca

**Keywords:** grassland, woody plant encroachment, biophysical variation, spectral separability, seasonal variation

## Abstract

Woody plant encroachment (WPE), the expansion of native and non-native trees and shrubs into grasslands, is a less studied factor that leads to declines in grassland ecosystem health. With the increasing application of remote sensing in grassland monitoring and measuring, it is still difficult to detect WPE at its early stages when its spectral signals are not strong enough. Even at late stages, woody species have strong vegetation characteristics that are commonly categorized as healthy ecosystems. We focus on how shrub encroachment can be detected through remote sensing by looking at the biophysical and spectral properties of the WPE grassland ecosystem, investigating the appropriate season and wavelengths that identify shrub cover, testing the spectral separability of different shrub cover groups and by revealing the lowest shrub cover that can be detected by remote sensing. Biophysical results indicate spring as the best season to distinguish shrubs in our study area. The earliest shrub encroachment can be identified most likely only when the cover reaches between 10% and 25%. A correlation between wavelength spectra and shrub cover indicated four regions that are statistically significant, which differ by season. Furthermore, spectral separability of shrubs increases with their cover; however, good separation is only possible for pure shrub pixels. From the five separability metrics used, Transformed divergence and Jeffries-Matusita distance have better interpretations. The spectral regions for pure shrub pixel separation are slightly different from those derived by correlation and can be explained by the influences from land cover mixtures along our study transect.

## 1. Introduction

Grasslands occur all over the world, extending from the Asian steppe, the Australian grasslands and the European grasslands, to the African savannas, the North American Great Plains and the South American Pampas. They offer a multitude of ecosystem services, such as forage for livestock, energy (e.g., biofuels, wind), carbon sequestration, water supply, recreational space, biodiversity preservation, food (e.g., beef), tourism, and genetic libraries (i.e., germplasms for future crops, ornamental plants) [1], hence they have high economic value (e.g., $1204 million/year to $2056 million/year for temperate grasslands [2]). However, nearly half (49.25%) of the global grasslands are degraded [3], predominantly due to overgrazing, intensive agricultural practices and climate change. One of the consequences leading to a global decline in grassland ecosystem health is woody plant encroachment (WPE), the expansion of native and non-native trees and shrubs into grasslands [4,5]. It is related to alterations in grassland primary productivity, nutrient cycling [6], biodiversity [7], structure and function [8], energy flow [9], and rangeland management [10]. Therefore, it is critical to detect WPE as early as possible to facilitate grassland management.

Woody plant encroachment is less studied with remote sensing methods because of several challenges. First, grasslands might appear in various WPE stages (i.e., early, moderate, or advanced), resulting in different woody cover within an image pixel [11]. The spectral signatures of woody plants may not be detectable at an early encroachment stage. Grasslands with WPE are highly heterogeneous and include land cover types that are, in many cases, smaller than the spatial resolution of medium-resolution remote sensors (10–100 m), especially during early encroachment. When the pixel size at which one studies WPE is coarser than the woody plant stand, a mixed pixel that includes various types of cover (e.g., woody plant, grass, bare ground, rock) occurs. Even though this has been recognized as a challenge, to our knowledge, no minimum WPE detection threshold has been established for grassland areas.

Second, a woody plant has typically healthy vegetation spectral features that are hard to separate from healthy productive grass species. Nevertheless, these two lifeforms differ in their biochemical and biophysical aspects, such as pigment concentration, water content, leaf surface, leaf internal structure, leaf thickness; which define their optical properties. Spectral absorption or reflection regions that are related to woody plants biochemical characteristics, such as lignin, nitrogen, chlorophyll, and water content could be useful towards their detection. For instance, it has been shown that chlorophyll and carotenoid content of woody species is higher than for grasses [12,13]. Since the visible portion of the electromagnetic spectrum is highly related to leaf pigment concentration, the reflectance in the green and absorption in the blue and red wavelengths might prove important when separating woody vegetation and grasses. Multispectral indices related to greenness and moisture are also important for WPE detection, as both of these could be higher for woody plants [14,15]. As for leaf structure (i.e., mesophyll structure, leaf thickness, leaf surface), there might be a difference in the reflectance of the leaves of woody species (dicotyledonous leaves) and grasses (monocotyledonous leaves) due to their different mesophyll structure [16,17], such as higher reflectance for the dicotyledonous leaves [18]. The Reflectance in the near infrared (NIR) region is mostly related to leaf structure. However, since remote sensors usually acquire data at the canopy and landscape scale, there is a difference in spectral response compared to the leaf scale. Factors that affect reflectance at that scale are related canopy architecture, such as leaf angle distribution, density, biomass, and leaf area index (LAI). Leaf orientation in broad leaf plants (e.g., shrubs) is more horizontal/planophilic, whereas grasses have more vertical orientation (erectophilic) [19]. Plants that are more planophilic tend to reflect more light upward than those that are more erectophilic [20], and this is more evident in the NIR region [21]. These leaf geometries can also be related to differences in LAI [22]. Therefore, we would like to see if these differences are evident in the biophysical and spectral properties of a WPE grassland.

Third, depending on the season of the study application, different indices and spectral regions seem to be important for shrub detection. For instance, hyperspectral indices related to greenness (e.g., Derivative Green Vegetation Index—DGVI) have better results during active woody plant growth, whereas those related to non-photosynthetic vegetation (e.g., Chlorophyll Absorption in Reflectance Index—CARI) perform better during senescence [23]. Woody plants and grasses might have a different phenology pattern, resulting in different spectral responses. Therefore, it is necessary to define the optimal woody plant detection timeframe within the growing season. This might not be important when using high-resolution spatial sensors, for which structural characteristics are used in combination with object-based methods [24]. However, for medium-resolution sensors, spectral differences due to phenology or land cover must be used. One example is the use of spectral separability and seasonal data in a composite image for woody plant mapping by Somers and Asner [25]. The results of this study showed that the use of multi-temporal image composites enhanced the detection of woody species due to their phenology. Hence, one must take into account the season in which shrub cover is most apparent and in which its spectral response is separable from the surroundings.

Last, when thinking about spectral separability, hyperspectral sensors (both spaceborne and airborne) have been widely used to detect WPE because of the advantages that their wide band range offers [25,26]. Specifically, with the use of hyperspectral data it is easier to find appropriate wavelengths to distinguish chemical and physical plant properties. Therefore, hyperspectral sensors are preferred when monitoring physiological plant traits [27]. Hyperspectral benefits enhance even more when using time series, giving the opportunity to explore phenological differences between grassy and encroaching vegetation [25]. Field-based hyperspectral measurements offer the opportunity to fine-tune spaceborne and airborne sensors for larger-scale shrub mapping. This involves the selection of appropriate spectral bands and regions for shrub detection with spectral separability metrics and statistics [25] (e.g., InStability Index, Transformed Divergence, etc.). Afterwards, one can define remote sensing indices that use these bands and apply a broader land cover classification procedure.

Based on the above, the overall goal of this study is to derive the season and sensitive spectral regions for shrub detection in grasslands. Our main objectives are (1) to understand the biophysical and spectral properties of the grassland ecosystem that undergoes WPE, (2) to investigate the appropriate seasons and wavelengths to identify shrub cover, (3) to test the spectral separability according to shrub cover, and (4) to reveal the lowest shrub cover that can be detected by remote sensing.

## 2. Study Area

**Figure 1:**
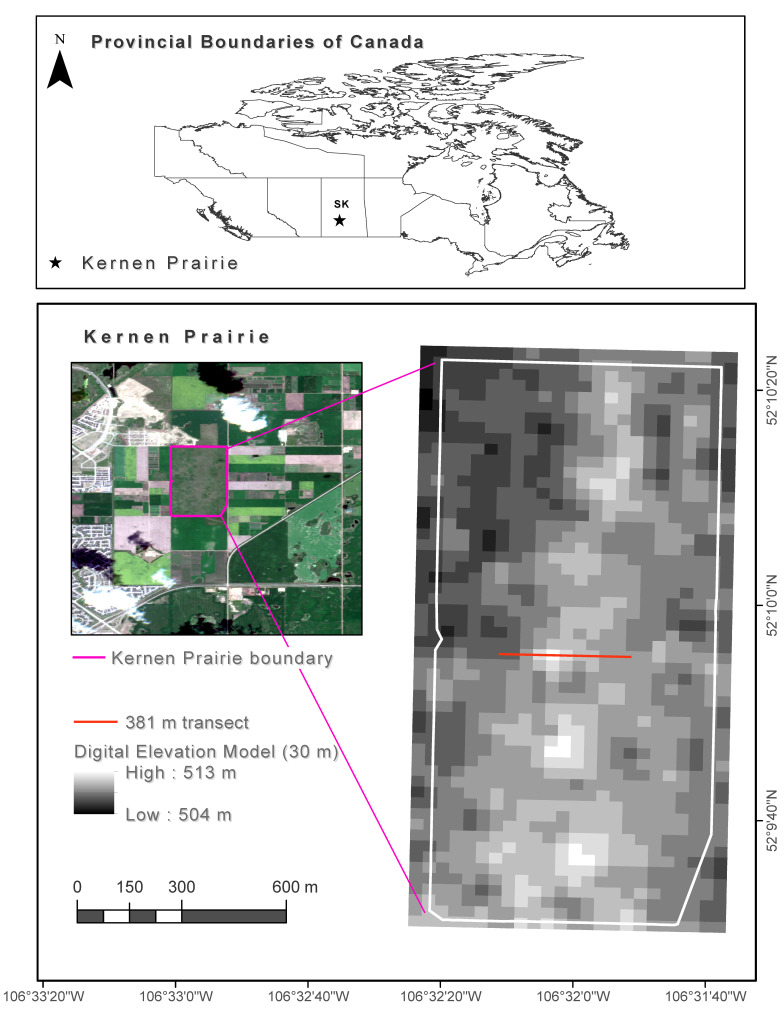
Location of Kernen Prairie within the provincial boundaries of Saskatchewan (SK), Canada (upper figure), on a Sentinel-2 image of 11 July 2020 (lower left figure), together with a detailed map of Kernen Prairie and the field transect location (lower right figure). Source of Canadian Provincial Boundaries: Statistics Canada (Open-Government License – Canada) [36], source of Sentinel-2 image: ESA (‘Copernicus Service information 2020’ for Copernicus Service Information) [37], source of digital elevation model: Shuttle Radar Topography Mission 1 Arc Second Global (National Aeronautics and Space Administration (NASA) and National Geospatial-Intelligence Agency (NGA) [38], source of Kernen Prairie land cover layers: Department of Plant Science, University of Saskatchewan.

The study area is the University of Saskatchewan’s Kernen Crop Research Farm in which WPE is an issue in its prairie stand. This area has a native remnant fescue prairie with common mixed prairie species which spans over 1.3 km^2^ at about 8 km NE of Saskatoon in Saskatchewan (52°10″ N, 106°33″ W, 510 m mean elevation) [28,29] (Figure 1). This site is in a transitional zone between the moist mixed grassland ecoregion (to the south) and aspen parkland (to the north). Mixed prairie graminoids are more common on drier sites, whereas fescue prairie graminoids are more apparent on mesic low topography sites [28,30]. This site was chosen as representative of a grassland ecosystem and could be easily accessed during the pandemic restriction.

Common grasses in the area are plains rough fescue (*Festuca altaica* subsp. *hallii*) (dominant grass), which grows together with slender wheatgrass (*Elymus trachycaulus* spp. *Trachycaulus (Link.)* Gould ex Shinners) and short bristle needle and thread grass (*Hesperostipa curtiseta* (Hitchc.) Barkworth) (sub-dominants). Frequent forbs are northern bedstraw (*Galium boreale*) and pasture sage (*Artemisia frigida*). Further, scattered patches of shrubs of various densities in the lower dry and saline parts of this site consist of western snowberry (*Symphoricarpos occidentalis* Hook.), wolf-willow (*Elaeagnus commutata* Bernh. ex Rydb.), and wild prairie rose (*Rosa arkansana*) [30,31]. At the lower moist land of Kernen Prairie, aspen stands can be found [32]. This site also has two invasive grasses, namely smooth brome (*Bromus inermis*) around the edges of the site which spreads towards the center, and Kentucky bluegrass (*Poa pratensis*) [31]. Variables that contribute to the plant community structure are related to landscape structure, such as slope, soil moisture, soil water availability, light availability [30], as well as fire and grazing regimes. In this study, we focus on two shrub species, western snowberry and wolf-willow that are encroaching species in the area.

The area has small slope variations without large soil temperature differences [30]. It has orthic dark brown chernozems soils of the Bradwell association which are loamy to fine sandy loam textured; it also has soils of the Sutherland association, which have a clay to clay-loamy texture [33]. These seem to have developed on the fine-textured lacustrine deposits of the former glacial Lake Saskatoon [30]. The regional climate of this area is categorized as semi-arid to dry subhumid according to the Thornthwaite classification [34]. Kernen prairie has a mean annual temperature of 3.3 °C, with a mean annual minimum temperature of −18.9 °C in January, and a mean maximum of 25.7 °C in July. Further, the mean annual precipitation is 340.4 mm [35].

The land cover types surrounding Kernen Prairie are cultivated lands and roads [28]. This area has been grazed or hayed sporadically until 1967 [32] and has never been ploughed or grazed heavily [29]. From 1986 and onward, a number of prescribed burns have been completed (to control the invasion of smooth brome, and shrub encroachment [29]), and other areas have been protected from fire for more than at least 105 years [28]. Further, there is a well in the southwest corner of the prairie that waters livestock [28]. Current management strategies involve light grazing by cattle from May to September (since 2006 until present) [31] and infrequent spring and fall patch burning [28].

## 3. Methods

**Figure 2:**
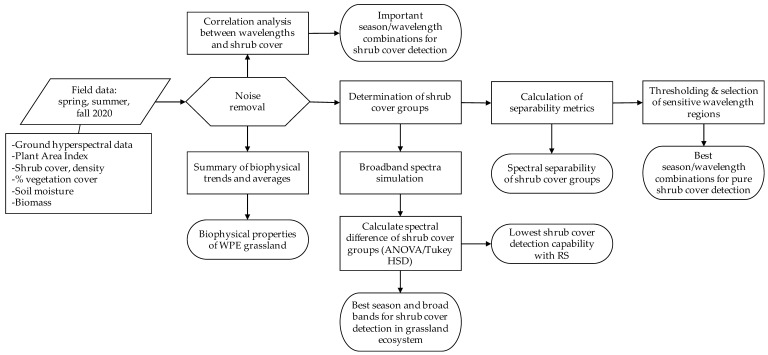
Conceptual model of methods that includes the input data, processing steps, and output data (WPE—Woody Plant Encroached, HSD—Honestly Significant Difference, RS—Remote Sensing).

The conceptual model of the methods that we followed in this study can be found in Figure 2.

### 3.1. Field Design and Data Collection

#### 3.1.1. Field Design

**Figure 3:**
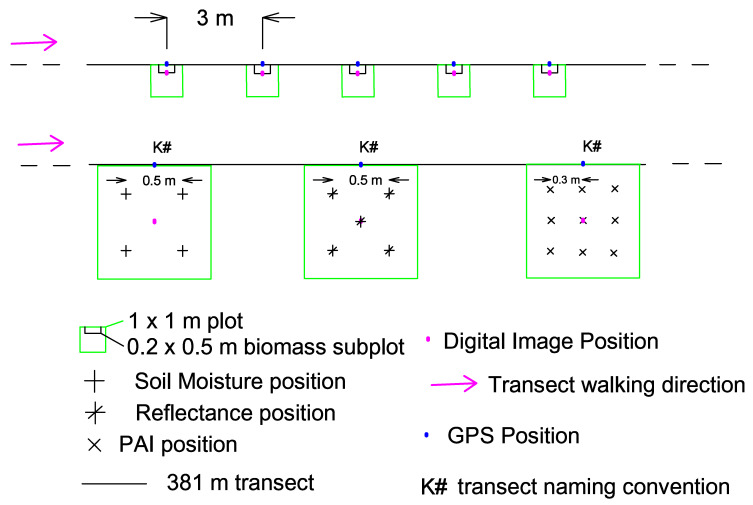
Field transect design and plot quadrat measurements (PAI—Plant Area Index, GPS—Global Positioning System).

A 381 m long transect was established in the study site along which 128 quadrats were placed at 3 m intervals (Figure 3). This transect was located along the main elevation variation gradient of Kernen Prairie (i.e., from 507 to 512 m, and then to 509 m), which corresponds to the East-West direction (Figure 1). About 40% of the transect (western side) lies on shallow clayey orthic dark brown Sutherland soil, and about 60% (eastern side) on discontinuous silty orthic dark brown Elstow soil [39]. The quadrats were 1 × 1 m and were placed tangent to the southern side of the transect line. This avoided trampling and facilitated reflectance measurements based on illumination conditions. Transect design has been used in a multitude of woody encroachment studies for field measurements (e.g., [5,40,41,42]. It makes sampling efforts faster and easier to establish, and it simultaneously captures the small-scale heterogeneity of the area. The dominant spatial scale in grasslands is between 0.2 and 1.5 m^2^, which is consistent with the biotic mechanisms of its species [43]. Therefore, cover estimates were recorded in 1 × 1 m quadrats to be representative of the scale of the plant community structure.

#### 3.1.2. Data Collection

We collected field data three times in the growing season (spring, summer, and fall). The data collected in 1 × 1 m quadrats along the transect included functional vegetation cover, ground hyperspectral data, plant area index (PAI), biomass, soil moisture, and landscape structure (Figure 3). In addition, we collected shrub cover and density with the line intercept transect (LIT) method in spring. Digital images were collected at nadir view above the center of each quadrat and 3D coordinates of each were obtained in UTM13N with the use of a Differential Global Positioning System with positional accuracy of less than 1 m.

Two different methods were used to determine shrub cover and density. The LIT method [40,41,42] and visual estimation within the transect quadrats. The LIT method is argued to be more efficient, since it covers a larger part of the study area and is fast. Shrubs for which their canopy falls on the transect line are recorded with their exact position (start and end along the transect); the sum of these lengths provide an estimate of the site’s shrub cover [44]. Thus, shrub cover from the LIT method is related to the total length of shrub cover along the transect, while it corresponds to the visual estimation of shrub cover within the quadrats. Shrub density was defined as the number of shrubs that fall along the transect for LIT and the total number of shrubs per quadrat.

Within each quadrat, we measured percentage ground cover for both the top layer (i.e., green grass, forb, shrub, standing dead vegetation) and the lower layer (i.e., moss, lichen, bare ground, litter, rock) through visual estimation based on the methodology proposed in Michalsky and Ellis [45]. This means that ground cover is approximated to the nearest 5% for cover between 10–90%, and to the nearest 1% for cover less than 5% and over 90%. The acquisition of % ground cover in each transect quadrat and season was conducted by the same observer so as to reduce potential observer bias.

We collected ground hyperspectral data from a spectroradiometer (ASD field-portable FieldSpec Pro, Malvern Panalytical Inc., Boulder, CO, USA) between 10:00 and 14:00 to maintain a stable ratio between diffuse and incoming solar radiation. The spectroradiometer collects between 350 and 2500 nm with a 1 nm band range. Reference measurements with a Spectralon panel were taken at least every 15 min. Two different measurements took place during the collection of such data. The first one included the collection of surface reflectance in five 0.5 m circular diameter areas in each 1 × 1 m plot, which were then averaged to the 1 × 1 m scale (Figure 3). In this case, the spectroradiometer was located at 1 m above ground, in nadir position, with a 25° field of view. The spectra collected with this method contain mixtures of all land cover types within each quadrat. The second type of measurements included the collection of shrub endmembers (i.e., wolfwillow and snowberry) that are encroaching in the study area. In this case, the spectroradiometer was placed closer to the shrubs and at least 10 samples for each shrub species were made to ensure the plant’s spectral variation was captured (i.e., leaves, branches). This allows us to have a spectral signature for ~100% shrub cover of the existing species in the study area.

Furthermore, we measured the PAI with the LAI 2000 plant canopy analyzer (LI-COR Inc., Lincoln, NE, USA) in every quadrat. We use the term PAI since we are measuring both green and non-green vegetation and both understory and overstory elements [46]. The smallest view cap (45°) was used to reduce errors of viewing both sparse and dense foliage, and to allow for correct below canopy reading averaging [47]. As for the appropriate number of below canopy readings, for grasslands, six below canopy readings have been used [48]. However, as shrub-encroached grasslands likely have higher heterogeneity, it was decided to use nine below-canopy readings to improve the spatial average. For each quadrat, PAI below-canopy readings took place at evenly spaced points (30 cm from the center) (Figure 3). Borders were avoided to eliminate adjacency effects. In addition, biomass was collected in a 0.5 × 0.2 m quadrat within each 1 × 1 m quadrat during the summer season. All existing vegetation was clipped, sorted into the functional vegetation cover classes (green grass, forb, shrub, dead, moss), dried at approximately 50 °C for a minimum of three days, and weighted with a scale of 0.1 g precision before and after drying. These samples were collected before the grazing season, and thus serve as a proxy of the annual net primary productivity of the site. For the subsequent fall measurements, the quadrat was moved south by 20 cm so that it did not include the biomass-clipped portion of ground. Lastly, we measured soil moisture with a Procheck pc1804 Soil Moisture (ICT International, Armidale, Australia) device in each quadrat at the same locations of the hyperspectral measurements (Figure 3), and we collected horizontal and vertical landscape structure (i.e., convex, concave, or linear) for each quadrat along the transect.

### 3.2. Data Processing

Averaging and noise removal: We averaged the hyperspectral, soil moisture, and PAI data to represent the 1 × 1 m quadrat and scaled the dry biomass up to 1 m^2^. We also removed outliers that fell outside of three standard deviations from the mean for the seasonal land cover estimations and PAI to eliminate noise from potential seasonal quadrat shifting. For all collected spectral data, we removed the water absorption regions between 1350–1430 nm, 1750–1980 nm, and 2330–2500 nm to reduce the noise.

Calculation of shrub cover groups: Dividing the quadrats into 5% shrub cover classes (e.g., 0.1–5%, 5.1–10%, 10.1–15%, etc.) gives us between 14 and 16 classes for all seasons. Since the spectral differences between those classes might not be significant, and because there is a small number of quadrats in each class, we decided to separate our data into meaningful clusters of shrub cover based on their spectral similarities. We only found western snowberry and prairie rose in the 128 transect quadrats. Prairie rose appeared in very low percentages (4.8% per quadrat on average for all seasons). Therefore, our first cluster includes the quadrats with 0% shrub and our last cluster includes the quadrats that correspond to the western snowberry endmembers (~100% shrub cover). To determine the major spectral shrub clusters for intermediate shrub cover (i.e., between 1% and 99%), we used cluster analysis. Cluster analysis divides the data into groups (clusters) that are more spectrally similar to each other than the data in other clusters [49]. In detail, we examined one centroid-based clustering method (K-means) and one hierarchical clustering method (Ward’s) [50,51]. K-means forms clusters around the centroids [51], while Ward’s clustering generates clusters that minimize the within-cluster dispersion at each binary split of the produced dendrogram [50].

As input data for cluster analysis, we used the 128 noise removed averaged hyperspectral measurements for each quadrat. There are a number of methods and statistics to determine the optimal number of clusters based on the data. We used the “Nbclust” R package to calculate 23 separate indices that generate the optimal number for each clustering method and growing season [52]. We used 2 and 15 clusters as minimum and maximum number of clusters and Euclidean distance for the calculation of the distance matrix each time. Finally, we selected the optimal number for each season and clustering method based on the majority rule of those indices. The clustering results gave us an initial idea about the data groupings from which we defined breakpoints that resulted in two alternative clustering schemes, one for each clustering method. We selected the final clustering based on the most balanced number of quadrat measurements for each shrub cover cluster.

### 3.3. Seasonal Variation of Biophysical Measurements

We hypothesize the season in which shrub cover is most apparent is the season in which its biophysical variables are strongest. To reach to this conclusion, we examined the seasonal trends of each variable. We subtracted each biophysical measurement (i.e., percentage cover, PAI, and soil moisture) for each land cover class between seasons (i.e., summer—spring and fall—summer). When the result of the subtraction is zero, it means that the biophysical variable for that quadrat remained stable. If the result is positive, we have an increase, and if negative, we have a decrease. We also looked at their seasonal averages by calculating the average biophysical measurements of each land cover class per season.

### 3.4. Correlation Analysis between Wavelengths and Shrub Cover

We examined which wavelengths have higher correlation with the visually estimated shrub cover measurements along the transect. To do this, we calculated the correlation between each wavelength and visually estimated shrub cover along the total transect (sample size = 128) per season. To determine which correlation method to use, we examined the assumptions of normality in the data for the shrub cover estimation. Visually analyzing the density plot and the normal quantile-quantile plot indicated data in non-normal distribution. We further generated the scatterplots between each wavelength and shrub cover estimate for all 128 quadrats, which confirmed that there is no strong linear relationship. We therefore implemented the Spearman’s rank correlation, which is a non-parametric method that measures the strength and direction of any monotonic (instead of linear) relationship between the ranked selected wavelength and visually estimated shrub cover [53]. We further identified the critical value of the Spearman’s rank correlation coefficient, r, for a two-tailed probability of a = 0.05 based on Zar [54]. To have a better overview of which wavelength regions have a statistically significant correlation with shrub cover along the transect, we plotted all correlation coefficients along the wavelength spectrum for each season.

### 3.5. Shrub Cover Spectral Separability Analysis

#### 3.5.1. Calculation of Separability Metrics

For this step we grouped our hyperspectral data for each season based on the cluster analysis and calculated the spectral separability between 0% shrub cover and each of the remaining clusters for every existing wavelength. We did this to see if the spectral separability changes with increasing shrub cover. Several separability metrics calculate how separable two clusters are. We used five different univariate statistic methods that are provided in the “separability” function of the “spatialEco” package in R [55]. These include the M-Statistic (M) [56], Bhattacharyya distance (B) [57], Jeffries-Matusita (JM) distance [58], Divergence [59,60], and Transformed Divergence (TD) [61]. They can provide the discrimination ability of each individual wavelength without taking into consideration their potential correlation [62]. This is important, since there might be autocorrelation along the transect.

The M-statistic is calculated by taking the difference of the means of the two clusters we are comparing normalized by the sum of their standard deviations. There is separation for M > 1, and poor separation for M < 1 [56]. The D statistic, defines the difference between two distributions by looking at the difference in mean values of the log-likelihood ratio [63]. The limitation of this statistic is its difficulty in interpretation as there is no upper limit, and its value continues to increase as two distributions separate [64]. To overcome this issue, the TD [61] scales the divergence statistic between 0 and 2, with 2 offering maximum separability. The B distance measures the divergence between two clusters by calculating the cosine of the angle between them [57]. Kailath [63] found that the B distance is easier to interpret than the D statistic, and that this metric performs equally or better than D. However, it has no upper limit (similar to D). Therefore, the JM distance was created to transform the B distance to a range between 0 and 2, with 2 suggesting maximum separability [61,65]. It is said that the JM distance can reduce the high separability features while enhancing the lower separability [62].

A normality check was performed to the wavelengths of each cluster. We performed a statistical test (Shapiro-Wilk’s [66]) before running the separability analysis. The Shapiro-Wilk’s test is considered a more powerful method over other statistical tests of normality [67] and has been used in similar spectral separability studies [68]. However, since its power might be lower with a small sample size (e.g., below 30), we decided to use additional visual methods (i.e., quantile-quantile plot, density plot). For all seasons, some clusters were normal along the whole wavelength spectrum and others were normal for 84–99.9% of the wavelengths. Even though some shrub clusters are partially not normal, we do not consider this to be an issue for the current spectral separability analysis, as these individual wavelengths will be aggregated in later analysis and some might not be considered to contribute to the spectral separability. Furthermore, following a non-parametric approach for a small number of samples in each cluster could result in larger biases than the slight deviation from normality for at most 15% of the dataset.

#### 3.5.2. Thresholding and Selection of Important Wavelength Regions

To select the final wavelength regions that are sensitive to separating between shrub cover and background cover, it is necessary to identify cut-off thresholds for each of the separability metrics that were calculated. Overall, it is considered that TD provides good separability when it has values above 1.8 [69] or 1.9 [64], with 2 providing the optimal separation between clusters. Similarly, features with TD values between 1.5 and 1.8 or 1.9 give moderate separation, whereas those with values below 1.5 give poor separation [64,69]. We therefore consider this classification for our own results (Table 1). Given the fact that JM follows the same scale (between 0 and 2), we use the same threshold rules for this statistic. When M is >1 we consider that there is good separation [56]. Although for this statistic it is hard to define an intermediate separation level, since there is no upper limit. Similarly, it is hard to define thresholds for the B and D statistics, due to the fact that they continue to increase without upper bound. Therefore, these statistics can only give a general idea of the important contributing wavelength regions towards the separability of the two clusters under examination each time. The final wavelength regions for which both the TD and JM have values above or equal to 1.8 were considered for having good separation (ensemble approach). A similar ensemble approach was used for the moderate separability regions.

### 3.6. Broadband Simulation and Shrub Cover Spectral Difference

#### 3.6.1. Broadband Simulation

We resampled the seasonal quadrat spectra into the broadband Landsat 8, Sentinel-2A, and Sentinel-2B bands with the use of their spectral response functions, which were retrieved from [70,71]. We performed the broadband simulation within the “hsdar” package in R with the use of the “spectralResampling” function [72].

#### 3.6.2. Broadband Spectral Difference between Shrub Cover Groups

To determine if there is a significant difference between shrub cover groups in each season per simulated broadband, we performed multiple one-way ANOVAs. The results were significant for all seasons and broad bands. Therefore, we performed a Tukey Honestly Significant Difference (HSD) post-hoc test to determine which shrub cover groups were significantly different from each other depending on the season and band. Since we have six shrub cover groups for the spring and summer season and five for the fall season, we have fifteen adjusted *p*-values from the Tukey HSD post-hoc test per band for spring and summer and ten for the fall season. We report those results in a table with two levels of adjusted *p*-value significance; below 0.1, and below 0.05.

## 4. Results

### 4.1. Seasonal Variation of Biophysical and Spectral Measurements

*Land cover*: From the average land cover for each season, shrub cover shows higher visibility in spring comparing with other land cover components. This indicates that spring is the preferable period for shrub monitoring (Table 2). Moreover, during the transition to summer, green grass increases by about 9% for 63% of the transect quadrats, covering up parts of lower cover, such as litter, bare ground and rock (Table S1). In the transition from summer to fall, as the vegetation reaches senescence, we see a decline of about 7% and 1% in green grass and forbs respectively (Table 2). On the other hand, the standing dead cover increases by about 13% for 86% of the quadrats, covering up more parts of the lower layers of litter, and bare ground (Table S1). On average, the dominant grass along all quadrats was rough fescue, representing 86% of the total grass cover, whereas the remaining parts primarily included wheatgrass species. Some quadrats also included smooth brome and Kentucky bluegrass invasives.

*Seasonal PAI*: There is a 0.81 increase between spring and summer for about 87% of the transect quadrats, and a subsequent 0.69 decrease between summer and fall for around 74% of the transect quadrats (Table S2). This fluctuation seems to correspond with the increase in green grasses during the summer and their subsequent senescence in the fall.

*Seasonal soil moisture*: The average seasonal soil moisture along the transect goes in line with the expected precipitation patterns of the region [73], with an increase during the summer (around 4% for 88% of the transect) and early fall (around 3% for 54% of the transect) (Table S3). The soil moisture levels are between 15% and 19% (Table 2), which are towards the lower limit for silty and silty clay soils [74], upon which the transect is located [39].

*Biomass*: Non-photosynthetic vegetation takes up most (63.1%) of the average summer biomass, after which green grasses (18.5%) and shrubs (14.6%) contribute towards most of the remaining biomass. Forbs (3.2%) and mosses (0.6%) contribute the least.

**Figure 4:**
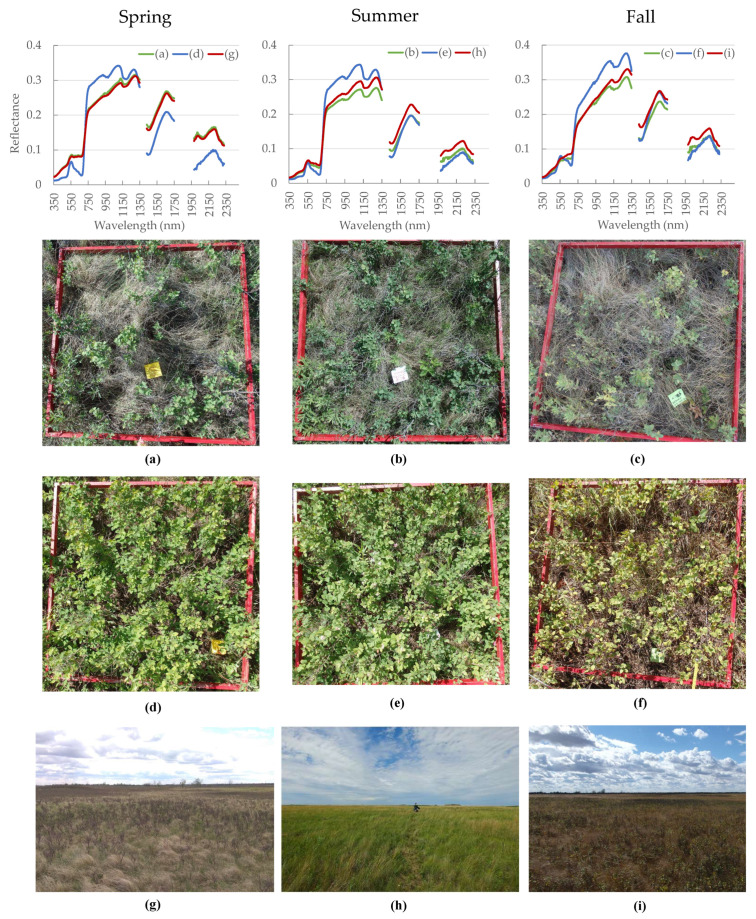
Spectral signatures that correspond to images (**a**–**i**) which are representative per season. Quadrat K36 with 25–45% shrub cover in (**a**) spring (9 June 2020), (**b**) summer (3 July 2020), and (**c**) fall (3 September 2020). Quadrat K96 with 60–80% shrub cover in (**d**) spring (11 June 2020), (**e**) summer (6 July 2020), and (**f**) fall (4 September 2020). Landscape pictures along the total study transect (128 quadrats) in (**g**) spring (27 May 2020), (**h**) summer (6 July 2020), and (**i**) fall (4 September 2020).

*Spectral*: When looking at the average spectral signature for all quadrats along the transect (Figure 4g–i), we can see an increase in chlorophyll absorption from the spring to the summer season for the red region of the spectrum (around 650 nm). On the other hand, the NIR remains fairly similar between those two seasons. In the fall season, we see a smooth increase in the visible portion due to the high amount of non-photosynthetic vegetation, and a lower reflectance along the NIR portion. The higher amount of vegetation moisture is responsible for larger absorption in the shortwave infrared (SWIR) region during summer, whereas the spring and fall seasons have a similar higher reflectance response in that region due to lower moisture.

Moreover, the LIT method reported 28.1% shrub cover along the transect for the spring season. Since the LIT method is purely quantitative, we consider it as a more precise estimate for shrub cover than the visual estimation inside the quadrats. The LIT method confirms the results from the visual shrub quadrat estimations with regards to shrub species contribution. Over the total transect area, we can find 1.1 western snowberry shrub, and 0.2 prairie rose per 1 m of transect during spring season, indicating the prevalence of western snowberry along the transect. A similar conclusion can be made when looking at the respective percentage cover for the shrub species along the transect (Table 3). Overall, the visual estimation of cover in the quadrats is underestimating prairie rose presence by 1.3% and western snowberry cover by 6.6%. Again, we trust the LIT values more, since the sample size covers the total transect; with 497 measurements (almost double) over 128 for each species in all quadrats.

Lastly, when looking at the increases and decreases in land cover (Table S1), the categories of “bare ground”, “rock”, and “other” remain stable for 96.8% of the quadrats across seasons. This indicates that the visual land cover estimation method is consistent and reliable across seasons and quadrats.

### 4.2. Relationships between Wavelengths and Shrub Cover

**Figure 5:**
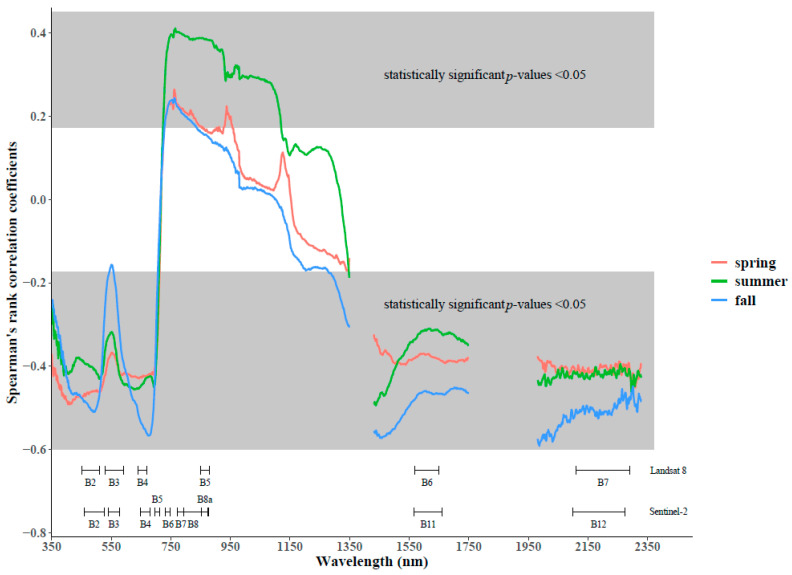
Spearman’s rank correlation coefficients for relationships between spectral reflectance and shrub cover measured during the 2020 growing seasons with corresponding Landsat 8 and Sentinel-2 bandwidths (source: ESA (‘Copernicus Service information 2021’ for Copernicus Service Information) [75], USGS (National Aeronautics and Space Administration (NASA)) [76]. The r-critical value for a two-tail test with a *p*-value of 0.05 was 0.17 and the spectral bandwidth is 1 nm.

There is clear variation in the strength of the relationship between shrub cover and spectral signals over seasons and wavelength (Figure 5). Specifically, the direction of the relationship differs in four regions of the spectrum between 350 nm and 2350 nm (those with *p*-values < 0.05). A negative relationship was found in the visible portion (between 350 nm and 700 nm), with more significant wavelengths around 420 nm (blue) for spring and summer, and around 495 nm (blue-green edge) and 680 nm (red) for fall. A positive relationship was found in the NIR portion (between 730 and 1120 nm), with more significant wavelengths around 760 nm for all seasons, which is stronger for the summer. Further, a negative relationship was found for all wavelengths above 1430 nm (SWIR region), with more significant wavelengths around 1430 nm for summer and more so for fall; and around 2000 nm for fall.

Within the visible region, the negative correlation (between −0.48 and −0.47) for all seasons in the blue region (around 420 nm) is more significant during spring and fall than for summer. This could be related to the stronger chlorophyll absorption during summer. Similar patterns are observed for the blue-green (495 nm) and red (680 nm) regions, where the start of shrub senescence and decrease in chlorophyll absorption leads to stronger negative correlations during fall (−0.51 and −0.56 respectively). The green peak (around 550 nm) is clearly less significant for all seasons and more so in the fall due to the lower chlorophyll content. The positive correlation in the NIR region (around 760 nm) is higher in the summer (around 0.39) and can be related to the higher reflectance of shrubs due to the scattering of their internal leaf structure in that season. For the SWIR region, we see strong negative correlations (−0.49 and −0.56) around one of the main water absorption features (1430 nm) during summer and fall respectively, and less stronger ones during spring (−0.33). This might be related to the increase in water holding capacity for shrubs during fall, when their transpiration is lower than summer and spring [77,78], compared to grass species. This can also be explained by the average increase in soil moisture from spring to fall along the quadrat (see Section 4.1). Lastly, in the far SWIR, we see the strongest negative correlation (−0.57) around 2000 nm for the fall season, which could again be explained by the higher water holding capacity of shrubs during fall.

### 4.3. Shrub Cover Spectal Separation Groups

We used the k-means and Ward’s clustering to group the transect quadrats in shrub cover percentage categories/groups for the spring and summer season, whereas the k-means and Ward’s clustering generated the same result for the fall season (Table 4).

**Figure 6:**
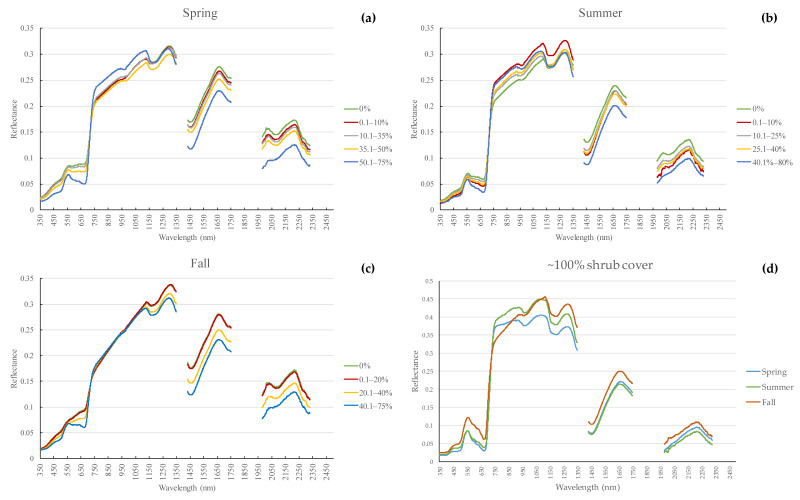
Average spectral response for shrub cover groups in (**a**) spring, (**b**) summer, (**c**) fall, and (**d**) 100% shrub cover over the three seasons ((**d**) was presented in a separate figure to remove its overshadowing effect).

The groups generated for each season are slightly different and are based on similarities in reflectance within each group. One can see the average spectral reflectance for all groups (except the ~100% shrub cover) in Figure 6a–c. There is a lower number of shrub cover percentage groups for the fall season, indicating that the groups are being separated into broader classes than for the spring and summer season. This means that these categories become more similar to each other and are harder to differentiate. This is reasonable, because all vegetation cover classes tend to have the same spectral response at the end of the growing season due to browning and senescence.

In spring (Figure 6a), the reflectance lowers in the visible spectrum (350–700 nm) as we move from 0% to 75% shrub cover, with only the 50.1–75% shrub cover group showing a distinct chlorophyll absorption in the red region (around 680 nm). In the NIR (700–1350 nm) the highest shrub cover group (50.1–75%) shows the highest reflectance. The shrub cover groups between 0% and 35% show similar reflectance, which is higher than the 35.1–50% shrub cover group. This perhaps is explained by the fact that the 0–35% shrub cover groups have, on average, higher forb and green grass cover (5.8% and 8% higher respectively). This could lead to higher reflectance than the 35.1–50% shrub cover groups, which are also affected by non-photosynthetic parts, such as branches and shadows. The two other parts within the SWIR region (1350–1750 nm and 1950–2350 nm) show a clear separation between all shrub cover groups; with a decline in reflectance as we move from 0% to 75% shrub cover.

In the summer (Figure 6b) there is a similar behavior as in the spring season for the visible spectrum. In the NIR we see a decline in reflectance as we move from 80% to 25% shrub cover, as expected. However, 0% shrub cover has a higher reflectance than the 0.1–10% shrub cover. When we examined the land cover estimations for each group, we saw that the 0.1–10% shrub cover quadrats have less green grass (2% less) and slightly more standing dead vegetation (0.3% more) and litter (0.3% more). These three land cover classes could be responsible for lowering the average reflectance of this shrub cover category. It becomes clear that the mixed pixel effect can have a major impact on shrub cover estimation. Along the two other parts of the SWIR region, we see a separation between shrub cover groups, which decline in reflectance when moving from 0% to 80% shrub cover. However, this separation is less clear than in the spring season for the intermediate groups (i.e., from 0.1% to 40% shrub cover).

During fall (Figure 6c), there is an increase for the lower shrub cover groups (i.e., 0% to 20% shrub cover) in the visible spectrum due to senescing grass (lower chlorophyll absorption). We also see an intermediate stage for the 20.1–40% shrub cover group, and a slight chlorophyll absorption still taking place around the red region (680 nm) for shrub cover between 40.1% and 75%. We see a collapse in spectral signatures in the NIR spectrum, at the end of which (1150–1350 nm) we see an inversion, with an increase in reflectance from 0% to 75% shrub cover. Since the 1150–1350 nm spectral range is used for estimation of vegetation water content [79], the reflectance for the higher shrub cover groups is lower along this part of the spectrum in comparison to the lower shrub cover groups. This is because the vegetation water content is much lower for the lower shrub cover groups (which contain mainly dry senescent grass). The differences in soil water content also play a major role here. For the SWIR region, there is also a decline in reflectance as shrub cover increases, with 0% and 0.1% to 20% shrub cover having almost similar reflectance.

When looking at the seasonal spectral response for the ~100% shrub cover group (Figure 6d), we see a fairly similar response in the visible spectrum between spring and summer. Summer has slightly higher reflectance. However, there is a clearly higher reflectance during fall. The increase in the visible spectrum during fall is due to a decrease in chlorophyll concentration. Along the NIR region, the reflectance is higher in summer than in spring and has similar absorption regions. Whereas, in the fall, reflectance increases between 700 and 950 nm, after which it has a similar reflectance as in summer (between 950 and 1150 nm), and the highest reflectance for the rest of the NIR spectrum. The higher fall reflectance between 1150 and 1350 nm is due to the lower vegetation water content compared to summer and spring. For the SWIR regions, fall has the highest reflectance due to the lowest amount of moisture absorption. Summer has the lowest reflectance, since it has the highest amount of moisture compared to the other two seasons.

### 4.4. Performance of Separability Metrics

In this section, we examine the shrub % cover group after which spectral separability between shrubs and the remaining land cover becomes possible for each season. After that, we make a comparison between the proposed wavelength regions from each separability metric threshold for the chosen shrub groups. Based on the ensemble results, we present the wavelengths regions most sensitive to shrub cover for each season.

**Figure 7:**
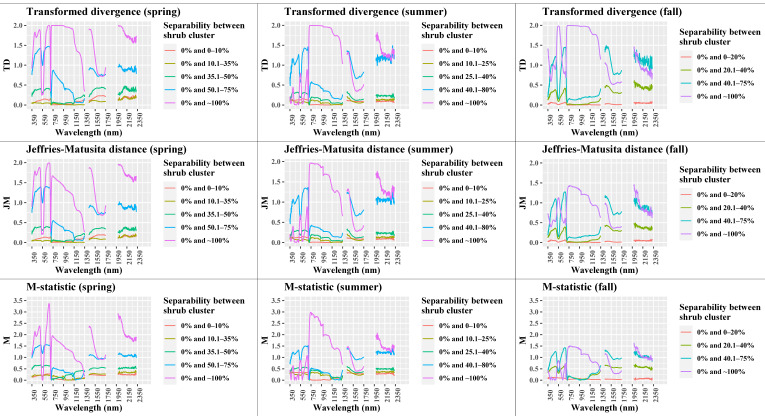
Seasonal separability metrics results of Transformed divergence, Jeffries-Matusita distance, and the M-statistic across all wavelengths for each defined shrub cover group.

Seasonal separability between shrub % groups: When looking at the separability metrics for each of the groups along the seasons (Figure 7, Figure S1), we can see that separability increases as the % of shrub cover in the group increases. We also see that separability is generally lower in the fall. TD and JM have fairly similar results, with JM having lower values for some wavelength regions in spring and summer, and for almost all higher shrub cover groups in fall. Moreover, the M-statistic also shows similar responses to the previous two, however on a different scale, where the higher values keep increasing, making the interpretation harder. The same holds for B and D (Figure S1). Based on the set thresholds for TD and JM (Table 1), none of the shrub groups between 0.1% and 80% cover for all seasons offer moderate or good separability, that is, above 1.5 (Figure 7). The only shrub group from which it is possible to differentiate from 0% shrub cover is the one that includes the endmember quadrats of ~100% shrub cover (pink line). In addition, the shrub group that belongs to a cover between 40.1% and 80% has a good separability for some wavelength regions according to the M-statistic. Fortunately, even with mixed pixels, there exist a number of spectral unmixing techniques that could enhance WPE mapping with coarser resolution pixels [80]. With spectral unmixing, each pixel gets assigned to fractions of its including classes, which are defined by endmembers [81].

As a next step, we classified the TD, JM, and M metrics for all seasons and groups based on the set thresholds. We selected those shrub groups that provide moderate or good separability and calculated the percentage of wavelength bands that contribute to each separability class (Table S4). The TD metric suggests higher number of wavebands that are important for separating shrub cover compared to the JM metric (24.1% more). Whereas, for the M metric, it is not possible to differentiate between moderate or good separation. It is clear that the spring season offers a higher number of bands with moderate and good separability across all three metrics (64.3% on average) compared to the summer and fall season (44.8% and 27.6% respectively). This is again an indication towards the preferable selection of the spring season for shrub monitoring.

Wavelength regions sensitive to shrub cover: To identify the wavelength regions that are sensitive to shrub cover for each season, we apply the ensemble method, where we select the TD and JM wavelengths that are classified as good or moderate under both metrics (Table 5, Figure 7). This separation holds only for differentiation between 0% and 100% shrub cover groups. The selected wavelength bands belong to certain spectral regions. Those that were below 10 nm wide were removed. The ensemble method could not be applied for the fall season, as the JM metric did not include any wavelengths in the moderate or good category. Therefore, we report the TD results for that season.

From the five spectral separability metrics, JM and TD allow for better interpretation and separation based on threshold establishment due to their upper limit (i.e., 2). In detail, the spring spectral regions in the blue (380–463 nm) and blue-green edge (467–509 nm) offer moderate and good separation of shrubs. This region is influenced by strong chlorophyll absorption [82]. The same holds for the red reflectance (604–617 nm—Moderate, 618–694 nm—Good), for which the red reflectance minimum (650–700 nm) offers the highest separation with values of TD and JM close to 2. Shrub species absorb more chlorophyll during springtime. Therefore, both blue and red allow for shrub differentiation from other background elements. On the other hand, the green peak (around 550 nm) is similar for both shrubs and background elements, and therefore not useful for shrub classification in spring. The NIR region seems to offer good separation according to the TD metric but only for a small moderate portion of the JM metric. However, the spectral signatures indicate a clear separation in that region, suggesting that the JM could be underestimating the separation potential in this case. Thus, JM tends to underestimate higher separability regions in some cases, confirming the findings of Gunal and Edizkan [62]. For the summer season, where the NIR values are about 0.05 units higher, JM is able to identify this region as important for good shrub separation. For the SWIR region we have separation in the near-SWIR (1431–1478 nm—Good). This region corresponds to the main water absorption region (between 1350–1450 nm), and to a region with rapid rise in spectra (1485–1518 nm—Moderate) that is sensitive to plant moisture [83]. It is clear that the shrub cover holds more moisture than the surrounding land cover, absorbing more in these spectral regions during spring. Furthermore, in the far-SWIR region, shrubs separate in a region related to water absorption (around 2050 nm) and cellulose absorption (around 2080 nm) (1981–2084 nm—Good) [84]. The shrub spectra have much lower reflection in this region due to their moisture content; whereas the rest of the land cover has higher non-photosynthetic content, thus higher reflectance, with an apparent absorption feature around 2080 nm. For the rest of the far-SWIR region (2105–2329 nm), shrub separation is moderate, with similarly lower reflectance due to the differences in moisture content and non-photosynthetic vegetation. There is a peak around 2250 nm for both categories, which is associated with differences in biomass [83].

In the summer season, other vegetation classes (grass, forbs) have also reached their peak in growth, thus separation in the visible bands of blue, green, and red is lower. However, the NIR region between 718–979 nm offers good separation. This is mainly due to the higher scattering of photons within the leaf structure of shrubs that lead to a higher reflectance in the NIR [82]. The near-SWIR region is no longer offering good separation, due to the overlap of the shrub spectral signature with other classes. However, the far-SWIR region between 1981–2061 nm offers moderate separation, which is mainly related to the differences in moisture absorption between shrub cover and the remaining land cover categories.

During fall, since the background vegetation is in senescence, the green peak within 525–579 nm stands out for the shrub cover that is still photosynthetically active (strong correlation with chlorophyll content) [82] and offers good separation. The declining slope that follows (580–597 nm) also offers moderate shrub separation. Since shrubs have not senesced yet during early fall, the NIR (704–1181 nm) and far-NIR (1183–1314 nm) regions remain important for good and moderate shrub separation due to higher biomass, PAI and plant density.

These results go in line with the indications from the M, B and D metrics. These show better separation between 0% and 100% shrub cover in the blue and red spectral regions for spring, the NIR for summer, and the green and NIR for the fall (Figure 6, Figure S1).

### 4.5. Broadband Simulation and Shrub Cover Spectral Difference

Broadband simulation: The mean values for each Landsat 8 and Sentinel-2A band per shrub cover group and season are presented in Table S5. The results for Sentinel-2B are very similar and are available in Table S6.

Broadband spectral difference between shrub cover groups: The Tukey HSD post-hoc adjusted *p*-values for each Landsat 8 and Sentinel-2A band per shrub cover group and season are presented in Table S7, and those of Sentinel-2B are available in Table S8. Several conclusions can be drawn from these results. First, we can see that it is not possible to detect any difference between groups 1 and 2 in any season. This means that it is impossible to detect shrub cover lower than 10% for the spring and summer, and lower than 20% for the fall season. Second, we see that the lowest possible shrub cover that is statistically different from other groups is between 10.1% and 25%, and that is during the summer season (Shrub group pair 1-3). Specifically, for the 90% confidence level (CI) of that pair, the SWIR 2 band of Landsat 8 and Sentinel-2 is significant. Similarly, the SWIR 2 band of Sentinel-2 is significant at the 90% CI for shrub cover between 10.1% and 35% during spring. Another observation that can be made, is that shrub cover groups that fall next to each other are for most seasons not separable when they have low shrub cover (e.g., shrub group pairs 1-2, 2-3, 3-4). On the other hand, they are more separable when they have higher shrub cover (e.g., shrub group pairs 4-5, 5-6). Lastly, when looking at differences between the extreme shrub cover groups of 0% and 100% (shrub group pair 1-6 for spring and summer and 1-5 for fall), we see that almost all bands show significant differences. However, the green and first red edge Sentinel-2 bands are not important during spring, and so are the blue bands for both sensors during summer and fall, indicating that these bands are not suitable for this case.

When looking at bands that are overall important for separating between shrub groups, we see that both the red and blue bands are the most important for separating between shrub cover groups during spring for both sensors. Further, the NIR band behaves poorly for both sensors, and so do the red edge and water vapor bands of Sentinel-2. The only case in which they are important, is for differences where the extreme shrub cover group is included (i.e., shrub cover group 6). Also, the SWIR 1 band has similar importance for the different shrub cover groups for both sensors. However, we see a difference in the behavior of the other bands between the two sensors for the spring season. Specifically, the SWIR 2 band of Sentinel-2 can separate a much larger number of shrub groups than the SWIR 2 band of Landsat 8 (11 vs. 5). In addition, the green band of Landsat 8 is able to separate between more shrub cover groups than the equivalent Sentinel-2 band (9 vs. 6). These are related to the different spectral response functions of the equivalent band in each sensor. The Sentinel-2 SWIR-2 band is slightly narrower than the respective Landsat 8 band (180 nm vs. 186.6 nm) [71], and the green Landsat 8 band is much wider than the Sentinel-2 band (57.33 nm vs. 35 nm) [70].

For the summer season, the SWIR-2 band for both sensors is the most important one at separating between shrub cover groups, followed by the green band. Overall, the visible bands (blue, green, red) are better at separating between lower levels of shrub cover groups (e.g., 1-4). Whereas, the NIR bands are better at separating higher shrub cover groups (e.g., 4-6, 5-6), and their behavior is similar for both sensors. Further, all red edge bands of Sentinel-2 have the same behavior as the NIR bands for both sensors and are only good at separating extreme shrub cover groups (e.g., 1-5, 1-6). The only exception is the red edge 1 band, which allows for separation between neighboring shrub cover groups (e.g., 4-5). The water vapor band is only capable of separating between groups that contain the highest shrub cover (i.e., group 6), and the SWIR-1 band behaves similarly poor for both sensors. It only separates between 4 shrub group pairs that have larger differences in cover (e.g., 1-5, 1-6).

In the fall season we see that the SWIR-2 and red bands are most important for both sensors at separating lower shrub cover groups. However, the red band of Sentinel is slightly stronger. It is the only band that can differentiate between the neighboring shrub covers of groups 3 and 4. The next most important band is the SWIR-1, which is similar for both sensors and offers differentiation between almost the same groups as the SWIR-2 band. The blue band is on a weaker side; however, it is still able to separate lower shrub cover classes, in which the Landsat sensor has a better performance than the corresponding Sentinel-2 band. Lastly, both green and NIR bands for all sensors and all red edge bands together with the water vapor band have a similar poor performance and are only able to separate pairs that include 100% shrub cover (i.e., group 5).

## 5. Discussion

Our results show that shrub cover is highest during the spring season. Homer et al. [85] also found slightly higher shrub cover in the spring season. Several studies take advantage of shrub phenology for their identification through remote sensing [25,86]. The spring season is in many cases chosen due to its match with the peak in growth for shrubs, when grasses have not reached their peak yet [23,24]. Our results go in line with this assumption, given the fact that the dominant shrub along our transect is Western snowberry, which’s leaves are fully expanded after mid to late May [87]. On the other hand, rough fescue cool-season grasses reach their peak of growth during late spring (late June) [88], hence, their cover is higher in the summer season (July). Furthermore, the seasonal fluctuations of other ephemeral cover (green grass, forbs, standing dead) follow known grassland patterns. Overall, it is known that the component of dead material and litter is high even during the growing season [89]. Specifically, a deep layer of litter and dead vegetation at the soil surface occurs due to the resistance of plains rough fescue to decomposition [90]. During fall, grasses, forbs, and shrubs start senescence, which explains the rise in standing dead cover. As new growth and dead material accumulates from spring to fall, the lower litter layers from the previous years become covered up; the same holds for bare ground and rock.

In this manuscript, we examined the relationship of various shrub cover percentages with spectral reflectance in three distinct ways. The correlations between transect shrub cover and the respective reflectance for the total wavelength spectrum gave an overall sense of the significant wavelength areas for each season. For the spectral separability, the only wavelength regions that were identified as good, are those that correspond to the separation of extreme groups (i.e., group 1 and 6, and 1 and 5). Therefore, these results can be compared with the respective broadband results for the pairs 1-6 in spring summer, and 1-5 in fall.

The correlation figure (Figure 5) showed higher correlation for the blue, NIR, and SWIR region in the spring, which matches the results of the good spectral separability and the broadband simulated significant differences between groups 1 and 6. However, the two latter also show that the red band is important. This can be explained, since for the extreme shrub cover group (group 6), the chlorophyll absorption in the red band is much stronger (and therefore more important), than it is for the lower shrub cover quadrats that are mixed with dead material, which are included in the correlation figure. Hence, this effect is not strong enough to appear in Figure 5. Overall, the blue and red regions are important for shrubs in this season due to strong chlorophyll absorption [82]. The position of the equivalent blue and red Landsat 8 and Sentinel-2 bands are able to capture this significant correlation with shrub cover.

In the correlation figure for the summer, we see a weaker significance for the visible portion, the highest correlation for the NIR and an equally important correlation for the SWIR 1 and SWIR 2 regions. Similarly, the visible wavelengths have lower separability between group 1 and 6 during summer, however the broadband simulation does include the red and green band. Nevertheless, their difference is not as good as the NIR region is for the separability and broadband simulation. Furthermore, there is agreement on the importance of SWIR 2 for separating between groups 1 and 6. This finding goes in line with another study, where the summer broadband SPOT 4 Normalized Difference Moisture Index (NDMI), which uses a combination of red and SWIR bands, had significant correlation (*p* < 0.01) with shrub biomass [91].

For the fall season, the correlation figure indicates important regions in the visible blue and red bands, a significant, but weaker than summer correlation for the NIR, and highest importance for both SWIR 1 and SWIR 2. However, when focusing on the differences between group 1 and 5 using spectral separability metrics and the broadband simulation, we see an almost opposite result, with green being the most significant region, followed by NIR, and a less important contribution from the SWIR region. In this case, the correlations in Figure 4 were not able to reflect the shrub cover dynamics but are rather related to the significant increases in the blue, red, and SWIR bands during the senescence of forbs and grasses in fall.

Overall, the correlation figure is able to detect the most dominant patterns during spring and summer but fails to indicate more subtle differences that are revealed by the other two methods. These are the importance of the red band during spring and the shrub contributing wavelengths during fall.

When looking at the broadband simulation results, it is possible to determine the overall importance of the sensor’s bands for separating between all potential shrub cover groups, apart from only the extreme ones that the separability method looks at. The bands that appear most frequently are the ones most sensitive to shrub cover changes. The visible bands are important at detecting differences between lower shrub cover groups. The NIR importance is higher during the summer season, but mostly for separating the highest shrub cover group (100%). This is because the NIR region is still very similar for intermediate shrub cover categories. Rather the short-wave infrared region, and in particular the far short-wave infrared region (SWIR-2) is good for lower shrub cover detection during summer and fall. These results show that the spectral absorption regions related to chlorophyll and water content are most useful towards shrub cover detection. This explains the successful use of spectral indices related to these two properties in other shrub detection studies (e.g., NDVI (Normalized Difference Vegetation Index), LWVI (Leaf Water Vegetation Index), GR (Green Ratio), NDMI) [23,24,91]. Overall, we can see that depending on the season, a different set of bands is more significant at separating shrub cover.

Even though the broadband simulation of field-based spectra shows potential for WPE detection in grasslands with certain band and season combinations, it is important to consider that these simulations do not represent satellite data conditions in their entirety. More specifically, satellite data are strongly affected by the atmosphere, and capture the land surface at a broader scale, in which topography can play an important role. Shadows and occlusions that are formed due to landscape relief lead to differences in vegetation reflectance and need to be accounted for. The direct solar beam and the diffuse skylight illumination both affect that reflectance [92]. Each slope and aspect of a terrain has an impact on reflectance and should be corrected with a model that can account for those factors over a composite sloping terrain [93]. For, these reasons, the current results should be cross-validated with satellite-based remote sensing data, such as Landsat 8 and Sentinel-2. We plan to implement this with future research that will establish specific narrowband hyperspectral indices and broadband multispectral indices optimally correlated with shrub cover along the study transect. To accomplish this, it is important to remove the potential spatial autocorrelation that exists between neighboring quadrats. This can be addressed by identifying the major scales of spatial variation in shrub cover with the use of wavelet analysis [94]. It will then be possible to select a satellite product with the optimal spatial and spectral scale for the detection of shrub cover in grasslands. Tests with satellite-imagery within the same and other study areas that cover different ecoregions and topographic conditions will be conducted and validated with field-derived woody cover.

## 6. Conclusions

This research was an investigation for shrub detection with a remote sensing approach and sheds more light on the seasonal variations in shrub cover and their respective sensitive spectral regions for shrub detection. We establish this with the use of field-based methods. Shrub cover appears highest during spring, and LIT proves to be superior for shrub cover estimation. The correlation between wavelength spectra and shrub cover shows four regions that are statistically significant, which differ by season. The separation of shrub cover measurements into groups based on spectral similarity showed that the spectral response of these groups becomes more similar during fall. Spectral separability of shrubs increases with cover; however, good separation is only possible for pure shrub pixels (~100%). There might be confusion between the spectral response of shrub cover and higher forb cover in the NIR region, whereas the SWIR region is not affected by such issues. From the five separability metrics used, TD and JM distance have better interpretation due to their upper limit. However, JM tends to underestimate the separability potential of some wavelengths during spring and summer. Overall, the spring season offered a higher number of bands that allow for moderate and good separation using both TD and JM compared to the other two seasons. Furthermore, based on the broadband simulated spectral differences, the earliest shrub cover can be separated when its cover reaches between 10.1% and 25% during summer and between 10.1% and 35% during spring. This is possible with the use of the SWIR-2 band of Landsat 8 and Sentinel-2. In addition, the shrub cover groups that fall next to each other are for most seasons not separable when they have lower shrub cover, whereas they become more separable for higher shrub cover. Common results from the three shrub detection techniques revealed significant relationships between shrub cover and the blue (spring), red (spring), NIR (stronger in summer), and far SWIR (summer and fall) spectral regions. These are spectral regions related to the differences in chlorophyll and water content between shrubs and their background land cover elements in grasslands. Cross-validation with satellite imagery is necessary to confirm the current results. To conclude, all seasons offer spectral regions that allow for good separation between shrub cover and background land cover. However, these regions are different in each season.

## Figures and Tables

**Table 1:** Separability threshold values (based on Kaufman and Remer [56], Campbell [69], and Bindel et al. [64]).

Separability Statistic	Threshold Value	Separability Class
M-Statistic	>1	Good
	≤1	Poor
Transformed Divergence and Jeffries-Matusita Distance	≥1.8	Good
	1.51–1.79	Moderate
	≤1.5	Poor

**Table 2:** Average seasonal variation of biophysical measurements per transect quadrat (%) (M—Mean value, SD—Standard Deviation, Min—Minimum value, Max—Maximum value).

		Spring			Summer			Fall		
		M	SD	Min–Max	M	SD	Min–Max	M	SD	Min–Max
**Cover (%)**	Green grass	25.5	8.6	5–65	30.2	7.7	5–55	23.5	6.3	10–40
	Shrub	**20.0**	19.3	0–75	18.0	17.2	0–80	17.4	16.5	0–75
	Forb	14.4	11.0	0–50	15.0	9.9	0–40	13.8	8.3	0–40
	Standing dead	30.5	11.3	0–60	30.4	9.0	0–50	41.9	12.8	0–80
	Litter	8.2	7.2	0–40	5.9	4.9	0–25	2.9	5.1	0–25
	Bare ground	0.7	3.1	0–25	0.3	2.2	0–20	0.1	1.3	0–15
	Rock	0.5	2.6	0–20	0.2	1.3	0–10	0.3	1.9	0–20
	Other	0.1	1.32	0–15	0.1	0.9	0–10	0.1	0.6	0–5
**PAI**		1.69	0.50	0.29–3.15	2.37	0.70	0.37–4.26	1.96	0.57	0.97–3.41
**Soil moisture (m³/m³)**		0.148	0.035	0.068–0.212	0.183	0.026	0.076–0.225	0.189	0.019	0.144–0.248
**Biomass (g/m²)**	Green grass				123.8	53.9	11–314			
	Forb				21.1	24.0	1–126			
	Shrub				97.5	139.0	1–888			
	Non-photosynthetic vegetation				422.8	194.1	84–931			
	Moss				3.8	7.3	1–40			
	Total				669.0					

**Table 3:** Average cover for shrubs and sub-species with the line intercept transect (LIT) method and the visual quadrat estimation for the spring season (W. Snowb.—Western snowberry, Prairie R.—Prairie rose, M-Mean, SD—Standard deviation).

	Average Shrub Cover (%)							Shrub Density Per 1 m		
	Total Shrub		W. Snowb.		Prairie R.		Total Shrub		W. Snowb.	Prairie R.
Estimation method	M	SD	M	SD	M	SD	M		M	M
LIT	28.1	-	25.4	-	2.7	-	1.3		1.09	0.23
Quadrat	20.2	19.2	18.8	19.1	1.4	2.2	-		-	-

**Table 4:** Final shrub percentage categories/groups for each season based on cluster analysis.

Season	Shrub Cover Groups (%)	Number of Quadrats per Group
Spring	0, <10, <35, <50, <75, <100	18, 35, 53, 12, 10, 32
Summer	0, <10, <25, <40, <80, <100	19, 35, 41, 22, 11, 11
Fall	0, <20, <40, <75, <100	19, 61, 38, 10, 20

**Table 5:** Shrub sensitive seasonal wavelength bands and spectral regions that offer moderate and good separation (B = Blue, G = Green, R = Red, NIR = Near infrared, SWIR = Shortwave infrared).

Season	Shrub Sensitive Wavelength Regions			
	Moderate		Good	
	Spectral Bands (nm)	Spectral Region	Spectral Bands (nm)	Spectral Region
Spring	380–466	B	467–509	B
	604–617	R	618–694	R
	723–883	NIR		
	1485–1518	SWIR-1	1431–1484	SWIR-1
	2105–2329	SWIR-2	1981–2104	SWIR-2
Summer	1981–2061	SWIR-2	718–979	NIR
	980–1122	NIR		
Fall	580–597	G	525–579	G
	1183–1314	NIR	704–1182	NIR

## Data Availability

The data presented in this study are available in this article and its Supplementary Materials.

## Supplementary Materials

The following are available online at https://www.mdpi.com/article/10.3390/s21093098/s1, Figure S1: Seasonal separability metrics results of Divergence (D) and Bhattacharyya distance (B), across all wavelengths for each defined shrub cover group, Table S1: Average seasonal land cover % change of transect quadrats, Table S2: Average seasonal Plant Area Index (PAI) change of quadrat cover, Table S3: Average seasonal soil moisture change within transect quadrats, Table S4: Wavelength classification according to separability thresholds for the seasonal Transformed Divergence (TD), Jeffries-Matusita (JM) and M-Statistic (M) metrics, Table S5: Mean simulated reflectance value (%) per Landsat 8 and Sentinel-2A band for each shrub cover group and season (B-Blue, G-Green, R-Red, RE-Red Edge, W. Vap.-Water Vapour), Table S6: Mean simulated reflectance value per Sentinel-2B band for each shrub cover group and season (B-Blue, G-Green, R-Red, RE-Red Edge, W. Vap.-Water Vapour), Table S7: Tukey Honestly Significant Difference (HSD) post-hoc adjusted *p*-values per Landsat 8 and Sentinel-2A band for each shrub cover group pair and season (B-Blue, G-Green, R-Red, RE-Red Edge, W. Vap.-Water Vapour). Red colored values are significant *p*-values within the 95% confidence interval (adj. *p*-value < 0.05) and yellow values are those that are significant within the 90% confidence interval, but not in the 95% confidence interval (adj. *p*-value between 0.05 and 0.1), Table S8: Tukey Honestly Significant Difference (HSD) post-hoc adjusted *p*-values per Sentinel-2B band for each shrub cover group pair and season (B-Blue, G-Green, R-Red, RE-Red Edge, W. Vap.-Water Vapour). Red colored values are significant *p*-values within the 95% confidence interval (CI) (adj. *p*-value < 0.05) and yellow values are those that are significant within the 90% CI, but not in the 95% CI (adj. *p*-value between 0.05 and 0.1).

## Abbreviations

B	Bhattacharyya distance
D	Divergence
JM	Jeffries-Matusita distance
LIT	Line Intercept Transect
M	M-Statistic
NIR	Near Infrared
PAI	Plant Area Index
SWIR	Shortwave Infrared
TD	Transformed Divergence
HSD	Honestly Significant Difference
WPE	Woody Plant Encroachment
