# Supplementary material for: Identification of the Optimal Season and Spectral Regions for Shrub Cover Estimation in Grasslands
Source: Sensors (Basel). 2021 Apr 29;21(9):3098. doi: 10.3390/s21093098 (PMC8124746; doi:10.3390/s21093098)
Supplement: Supplementary file 1 [file sensors-21-03098-s001.zip › Figure S1.pdf]

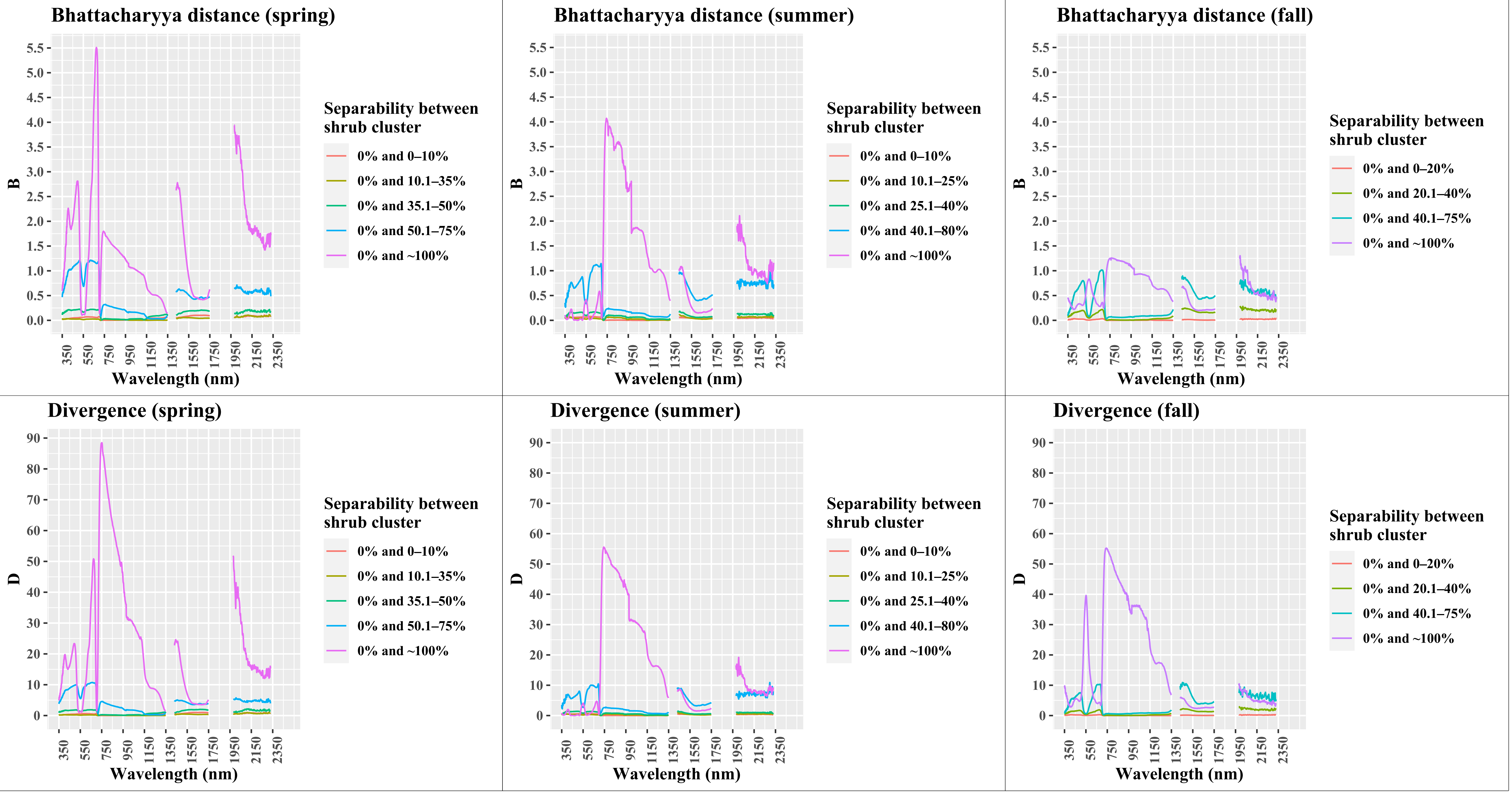

Figure S1.Seasonal separability metrics results of Divergence (D) and Bhattacharyya distance (B), across all wavelengths for each defined shrub cover group
