# Supplementary material for: Identification of the Optimal Season and Spectral Regions for Shrub Cover Estimation in Grasslands
Source: Sensors (Basel). 2021 Apr 29;21(9):3098. doi: 10.3390/s21093098 (PMC8124746; doi:10.3390/s21093098)
Supplement: Supplementary file 1 [file sensors-21-03098-s001.zip › Tables S1-S8.docx]

**Table S1.** Average seasonal land cover % change of transect quadrats.

|  | Spring to summer average change | | | Summer to fall average change | | |
| --- | --- | --- | --- | --- | --- | --- |
|  | **Stable** | **Increase** | **Decrease** | **Stable** | **Increase** | **Decrease** |
| Grass cover (%) | 0 | 9 | −7 | 0 | 7 | −10 |
| # quadrats | 33 | 80 | 15 | 22 | 14 | 90 |
| % quadrats | 26% | 63% | 12% | 17% | 12% | 71% |
| Shrub cover (%) | 0 | 6 | −7 | 0 | 6 | −6 |
| # quadrats | 43 | 33 | 46 | 48 | 34 | 43 |
| % quadrats | 35% | 27% | 38% | 38% | 27% | 34% |
| Forb cover (%) | 0 | 7 | −8 | 0 | 7 | −8 |
| # quadrats | 51 | 46 | 29 | 40 | 36 | 51 |
| % quadrats | 40% | 37% | 23% | 31% | 28% | 40% |
| Standing dead cover (%) | 0 | 8 | −9 | 0 | 13 | −5 |
| # quadrats | 30 | 48 | 47 | 11 | 107 | 7 |
| % quadrats | 24% | 38% | 38% | 9% | 86% | 6% |
| Litter cover (%) | 0 | 5 | −6 | 0 | 4 | −6 |
| # quadrats | 27 | 24 | 64 | 45 | 17 | 64 |
| % quadrats | 23% | 21% | 56% | 36% | 13% | 51% |
| Bare ground cover (%) | 0 | N/A | −5 | 0 | N/A | −8 |
| # quadrats | 107 | 0 | 8 | 125 | 0 | 3 |
| % quadrats | 93% | 0% | 7% | 98% | 0% | 2% |
| Rock cover (%) | 0 | N/A | −8 | 0 | 7.5 | −5 |
| # quadrats | 123 | 0 | 5 | 125 | 2 | 1 |
| % quadrats | 96% | 0% | 4% | 98% | 2% | 1% |
| Other cover (%) | 0 | N/A | −5 | 0 | 5 | −5 |
| # quadrats | 127 | 0 | 1 | 126 | 1 | 1 |
| % quadrats | 99% | 0% | 1% | 98% | 1% | 1% |

**Table S2.** Average seasonal Plant Area Index (PAI) change of quadrat cover.

|  | Spring to summer | | | Summer to fall | | |  |
| --- | --- | --- | --- | --- | --- | --- | --- |
|  | **Stable** | **Increase** | **Decrease** | **Stable** | **Increase** | **Decrease** | |
| PAI average change | 0 | 0.81 | −0.25 | 0 | 0.43 | −0.69 | |
| # quadrats | 0 | 108 | 16 | 1 | 32 | 94 | |
| % quadrats | 0% | 87.10% | 12.90% | 0.78% | 25.20% | 74.02% | |

**Table S3.** Average seasonal soil moisture change within transect quadrats.

| Soil moisture (m³/m³) | Spring to summer | | | Summer to fall | | |
| --- | --- | --- | --- | --- | --- | --- |
|  | **Stable** | **Increase** | **Decrease** | **Stable** | **Increase** | **Decrease** |
| Average change | 0 | 0.041 | −0.011 | 0 | 0.029 | −0.022 |
| # quadrats | 0 | 113 | 15 | 2 | 69 | 57 |
| % quadrats | 0% | 88.28% | 11.72% | 1.56% | 53.91% | 44.53% |

**Table S4.** Wavelength classification according to separability thresholds for the seasonal Transformed Divergence (TD), Jeffries-Matusita (JM) and M-Statistic (M) metrics.

| **Season** | **Separability metric** | **Separation between shrub groups** | **Separability class along the wavelength spectrum (%)** | | |
| --- | --- | --- | --- | --- | --- |
|  |  |  | **Poor** | **Moderate** | **Good** |
| **Spring** | **TD** | 0% and ~100 % | 32.2% | 22.9% | 44.8% |
|  | JM | 0% and ~100 % | 50.4% | 33.3% | 16.3% |
|  | M | 0% and ~100 % | 24.4% | 75.6% | |
|  |  | 0% and 50.1–75% | 50.6% | 49.4% | |
| **Summer** | TD | 0% and ~100 % | 59.0% | 13.6% | 27.4% |
|  | JM | 0% and ~100 % | 70.6% | 13.7% | 15.7% |
|  | M | 0% and ~100 % | 36.1% | 63.9% | |
|  |  | 0% and 40.1–80% | 53.3% | 46.7% | |
| **Fall** | TD | 0% and ~100 % | 58.3% | 9.6% | 32.0% |
|  | JM | 0% and ~100 % | 100% | 0.0% | 0.0% |
|  | M | 0% and ~100 % | 58.9% | 41.1% | |
|  |  | 0% and 40.1–75% | 64.9% | 35.1% | |

**Table S5.** Mean simulated reflectance value (%) per Landsat 8 and Sentinel-2A band for each shrub cover group and season (B-Blue, G-Green, R-Red, RE-Red Edge, W. Vap.-Water Vapour).

|  |  |  | **Mean simulated reflectance value (%)** | | | | | | | | | | | | | | | | |
| --- | --- | --- | --- | --- | --- | --- | --- | --- | --- | --- | --- | --- | --- | --- | --- | --- | --- | --- | --- |
|  | **Shrub cover group** | **Shrub cover %** | **Landsat-8** | | | | | | **Sentinel-2A** | | | | | | | | | | |
|  |  |  | **B** | **G** | **R** | **NIR** | **SWIR 1** | **SWIR 2** | **B** | **G** | **R** | **RE1** | **RE2** | **RE3** | **NIR** | **RE4** | **W. Vap.** | **SWIR 1** | **SWIR 2** |
| **Spring** | **1** | **0** | 5.6 | 8.3 | 8.8 | 23.7 | 25.3 | 15.9 | 5.9 | 8.4 | 8.8 | 12.4 | 19.6 | 21.6 | 22.9 | 23.7 | 25.1 | 25.5 | 16.0 |
|  | **2** | **0.1**–**10** | 5.4 | 8.1 | 8.4 | 23.8 | 24.4 | 15.0 | 5.7 | 8.2 | 8.4 | 12.0 | 19.7 | 21.8 | 23.0 | 23.9 | 25.1 | 24.6 | 15.2 |
|  | **3** | **10.1**–**35** | 5.3 | 8.1 | 8.4 | 24.1 | 24.0 | 14.6 | 5.6 | 8.2 | 8.4 | 12.1 | 20.1 | 22.2 | 23.4 | 24.2 | 25.6 | 24.2 | 14.7 |
|  | **4** | **35.1**–**50** | 4.7 | 7.5 | 7.4 | 23.3 | 23.1 | 13.8 | 5.0 | 7.6 | 7.4 | 11.3 | 19.5 | 21.4 | 22.6 | 23.3 | 24.9 | 23.3 | 14.0 |
|  | **5** | **50.1**–**75** | 3.4 | 6.4 | 5.2 | 26.0 | 20.9 | 11.3 | 3.7 | 6.6 | 5.1 | 10.3 | 21.9 | 24.2 | 25.3 | 26.1 | 27.2 | 21.1 | 11.5 |
|  | **6** | **100** | 3.0 | 7.5 | 3.6 | 38.8 | 19.6 | 9.8 | 3.3 | 7.9 | 3.2 | 12.3 | 34.5 | 37.8 | 38.3 | 38.8 | 38.3 | 19.9 | 8.5 |
| **Summer** | **1** | **0** | 3.9 | 6.7 | 6.1 | 23.7 | 21.6 | 12.3 | 4.2 | 6.9 | 6.0 | 10.2 | 19.4 | 21.7 | 22.9 | 23.7 | 25.1 | 21.9 | 12.4 |
|  | **2** | **0.1**–**10** | 3.6 | 6.3 | 5.5 | 24.0 | 20.6 | 11.3 | 3.8 | 6.5 | 5.4 | 9.8 | 19.5 | 21.9 | 23.2 | 24.0 | 25.2 | 20.8 | 11.5 |
|  | **3** | **10.1**–**25** | 3.7 | 6.4 | 5.6 | 25.1 | 20.6 | 11.1 | 3.9 | 6.6 | 5.5 | 9.9 | 20.5 | 23.1 | 24.3 | 25.2 | 25.9 | 20.8 | 11.2 |
|  | **4** | **25.1**–**40** | 3.4 | 6.1 | 5.1 | 25.6 | 20.1 | 10.6 | 3.7 | 6.3 | 5.0 | 9.5 | 20.8 | 23.5 | 24.8 | 25.6 | 26.5 | 20.4 | 10.7 |
|  | **5** | **40.1**–**80** | 2.7 | 5.4 | 3.7 | 26.7 | 18.1 | 8.9 | 2.9 | 5.6 | 3.5 | 8.5 | 21.8 | 24.7 | 25.9 | 26.7 | 27.3 | 18.3 | 9.0 |
|  | **6** | **100** | 3.8 | 7.7 | 4.5 | 42.0 | 19.2 | 7.2 | 4.1 | 8.0 | 4.2 | 12.3 | 35.3 | 39.8 | 41.1 | 42.0 | 42.1 | 19.5 | 7.3 |
| **Fall** | **1** | **0** | 4.9 | 7.5 | 9.2 | 21.4 | 25.7 | 15.4 | 5.2 | 7.5 | 9.4 | 12.4 | 16.4 | 18.3 | 20.2 | 21.4 | 24.2 | 25.9 | 15.6 |
|  | **2** | **0.1**–**20** | 4.8 | 7.3 | 9.0 | 21.6 | 25.5 | 15.1 | 5.0 | 7.3 | 9.2 | 12.3 | 16.5 | 18.4 | 20.4 | 21.6 | 24.5 | 25.8 | 15.3 |
|  | **3** | **20.1**–**40** | 4.2 | 6.9 | 7.8 | 21.8 | 22.8 | 13.2 | 4.5 | 6.9 | 7.8 | 11.6 | 16.9 | 18.8 | 20.7 | 21.8 | 24.4 | 23.0 | 13.4 |
|  | **4** | **40.1**–**75** | 3.5 | 6.5 | 6.2 | 21.8 | 21.1 | 11.6 | 3.8 | 6.7 | 6.1 | 11.0 | 17.2 | 19.0 | 20.7 | 21.8 | 24.2 | 21.3 | 11.8 |
|  | **5** | **100** | 5.0 | 11.4 | 7.4 | 38.4 | 22.6 | 9.7 | 5.6 | 11.8 | 6.7 | 18.0 | 31.8 | 34.9 | 37.0 | 38.4 | 40.6 | 22.9 | 9.8 |

**Table S6.** Mean simulated reflectance value per Sentinel-2B band for each shrub cover group and season (B-Blue, G-Green, R-Red, RE-Red Edge, W. Vap.-Water Vapour).

|  | Shrub cover group | Shrub cover % | Sentinel-2B mean simulated reflectance value (%) | | | | | | | | | | |
| --- | --- | --- | --- | --- | --- | --- | --- | --- | --- | --- | --- | --- | --- |
|  |  |  | **B** | **G** | **R** | **RE1** | **RE2** | **RE3** | **NIR** | **RE4** | **W. Vap.** | **SWIR 1** | **SWIR 2** |
| Spring | **1** | **0%** | 5.9 | 8.4 | 8.8 | 12.3 | 19.4 | 21.6 | 22.9 | 23.7 | 25.1 | 25.3 | 16.0 |
|  | **2** | **0.1**–**10%** | 5.7 | 8.2 | 8.4 | 12.0 | 19.6 | 21.7 | 23.1 | 23.8 | 25.1 | 24.4 | 15.1 |
|  | **3** | **10.1**–**35%** | 5.6 | 8.2 | 8.4 | 12.0 | 19.9 | 22.1 | 23.4 | 24.1 | 25.6 | 24.1 | 14.7 |
|  | **4** | **35.1**–**50%** | 5.0 | 7.6 | 7.4 | 11.3 | 19.3 | 21.3 | 22.6 | 23.3 | 24.9 | 23.1 | 14.0 |
|  | **5** | **50.1**–**75%** | 3.7 | 6.6 | 5.0 | 10.2 | 21.6 | 24.1 | 25.3 | 26.0 | 27.2 | 20.9 | 11.4 |
|  | **6** | **100%** | 3.3 | 7.9 | 3.2 | 12.1 | 34.0 | 37.7 | 38.3 | 38.8 | 38.4 | 19.7 | 8.4 |
| Summer | **1** | **0%** | 4.2 | 6.9 | 6.0 | 10.2 | 19.2 | 21.6 | 22.9 | 23.7 | 25.1 | 21.7 | 12.4 |
|  | **2** | **0.1**–**10%** | 3.8 | 6.5 | 5.4 | 9.7 | 19.3 | 21.8 | 23.2 | 23.9 | 25.2 | 20.6 | 11.4 |
|  | **3** | **10.1**–**25%** | 3.9 | 6.6 | 5.5 | 9.8 | 20.2 | 23.0 | 24.3 | 25.1 | 25.9 | 20.7 | 11.1 |
|  | **4** | **25.1**–**40%** | 3.7 | 6.3 | 5.0 | 9.4 | 20.5 | 23.5 | 24.8 | 25.6 | 26.5 | 20.2 | 10.7 |
|  | **5** | **40.1**–**80%** | 2.9 | 5.6 | 3.5 | 8.4 | 21.5 | 24.6 | 25.9 | 26.7 | 27.3 | 18.1 | 8.9 |
|  | **6** | **100%** | 4.1 | 8.1 | 4.2 | 12.1 | 34.8 | 39.7 | 41.1 | 42.0 | 42.2 | 19.3 | 7.2 |
| Fall | **1** | **0%** | 5.2 | 7.4 | 9.4 | 12.4 | 16.3 | 18.2 | 20.2 | 21.4 | 24.1 | 25.8 | 15.6 |
|  | **2** | **0.1**–**20%** | 5.0 | 7.3 | 9.2 | 12.3 | 16.4 | 18.3 | 20.4 | 21.6 | 24.4 | 25.6 | 15.3 |
|  | **3** | **20.1**–**40%** | 4.5 | 6.9 | 7.8 | 11.6 | 16.8 | 18.7 | 20.7 | 21.8 | 24.3 | 22.8 | 13.3 |
|  | **4** | **40.1**–**75%** | 3.8 | 6.7 | 6.1 | 10.9 | 17.0 | 18.9 | 20.7 | 21.8 | 24.2 | 21.1 | 11.7 |
|  | **5** | **100%** | 5.6 | 11.8 | 6.7 | 17.8 | 31.6 | 34.7 | 37.0 | 38.4 | 40.6 | 22.7 | 9.7 |

**Table S7.** Tukey Honestly Significant Difference (HSD) post-hoc adjusted *p*-values per Landsat 8 and Sentinel-2A band for each shrub cover group pair and season (B-Blue, G-Green, R-Red, RE-Red Edge, W. Vap.-Water Vapour). Red colored values are significant *p*-values within the 95% confidence interval (adj. *p*-value<0.05) and yellow values are those that are significant within the 90% confidence interval, but not in the 95% confidence interval (adj. *p*-value between 0.05 and 0.1).

|  |  | **Tukey HSD post-hoc adjusted *p*-values** | | | | | | | | | | | | | **Yell.** | **< 0.1** | **Red** | **< 0.05** |
| --- | --- | --- | --- | --- | --- | --- | --- | --- | --- | --- | --- | --- | --- | --- | --- | --- | --- | --- |
| **Season** | **Shrub group pairs** | **Landsat-8** | | | | | | **Sentinel-2A** | | | | | | | | | | |
|  |  | **B** | **G** | **R** | **NIR** | **SWIR 1** | **SWIR 2** | **B** | **G** | **R** | **RE 1** | **RE 2** | **RE 3** | **NIR** | **RE 4** | **W. Vap.** | **SWIR 1** | **SWIR 2** |
| **Spring** | **1-2** | 0.780 | 0.876 | 0.566 | 1.000 | 0.753 | 0.975 | 0.779 | 0.904 | 0.565 | 0.900 | 1.000 | 1.000 | 1.000 | 1.000 | 1.000 | 0.760 | 0.482 |
|  | **1-3** | 0.533 | 0.828 | 0.524 | 0.999 | 0.312 | 0.831 | 0.534 | 0.874 | 0.547 | 0.947 | 0.994 | 0.995 | 0.997 | 0.999 | 0.994 | 0.319 | **0.054** |
|  | **1-4** | **0.001** | **0.050** | **0.001** | 1.000 | **0.096** | 0.753 | **0.001** | **0.087** | **0.001** | 0.175 | 1.000 | 1.000 | 1.000 | 1.000 | 1.000 | **0.097** | **0.020** |
|  | **1-5** | **0.000** | **0.000** | **0.000** | 0.615 | **0.000** | **0.052** | **0.000** | **0.000** | **0.000** | **0.000** | 0.471 | 0.491 | 0.567 | 0.615 | 0.673 | **0.000** | **0.000** |
|  | **1-6** | **0.000** | **0.004** | **0.000** | **0.000** | **0.000** | **0.000** | **0.000** | 0.318 | **0.000** | 0.999 | **0.000** | **0.000** | **0.000** | **0.000** | **0.000** | **0.000** | **0.000** |
|  | **2-3** | 0.999 | 1.000 | 1.000 | 0.999 | 0.971 | 0.996 | 0.999 | 1.000 | 1.000 | 1.000 | 0.996 | 0.998 | 0.999 | 0.999 | 0.989 | 0.971 | 0.832 |
|  | **2-4** | **0.010** | 0.221 | **0.032** | 0.998 | 0.497 | 0.956 | **0.011** | 0.305 | **0.035** | 0.517 | 1.000 | 0.999 | 0.999 | 0.998 | 1.000 | 0.493 | 0.328 |
|  | **2-5** | **0.000** | **0.000** | **0.000** | 0.566 | **0.000** | 0.117 | **0.000** | **0.000** | **0.000** | **0.001** | 0.432 | 0.463 | 0.525 | 0.566 | 0.601 | **0.000** | **0.000** |
|  | **2-6** | **0.000** | **0.027** | **0.000** | **0.000** | **0.000** | **0.000** | **0.000** | 0.816 | **0.000** | 0.965 | **0.000** | **0.000** | **0.000** | **0.000** | **0.000** | **0.000** | **0.000** |
|  | **3-4** | **0.014** | 0.191 | **0.019** | 0.982 | 0.781 | 0.994 | **0.015** | 0.261 | **0.019** | 0.350 | 0.991 | 0.987 | 0.984 | 0.982 | 0.986 | 0.777 | 0.782 |
|  | **3-5** | **0.000** | **0.000** | **0.000** | 0.675 | **0.001** | 0.190 | **0.000** | **0.000** | **0.000** | **0.000** | 0.588 | 0.597 | 0.650 | 0.675 | 0.804 | **0.001** | **0.000** |
|  | **3-6** | **0.000** | **0.013** | **0.000** | **0.000** | **0.000** | **0.000** | **0.000** | 0.762 | **0.000** | 0.990 | **0.000** | **0.000** | **0.000** | **0.000** | **0.000** | **0.000** | **0.000** |
|  | **4-5** | **0.000** | **0.025** | **0.000** | 0.523 | 0.215 | 0.687 | **0.000** | **0.057** | **0.000** | 0.273 | 0.504 | 0.482 | 0.512 | 0.523 | 0.659 | 0.223 | **0.009** |
|  | **4-6** | **0.000** | 1.000 | **0.000** | **0.000** | **0.000** | **0.043** | **0.000** | 0.857 | **0.000** | 0.204 | **0.000** | **0.000** | **0.000** | **0.000** | **0.000** | **0.000** | **0.000** |
|  | **5-6** | 0.223 | **0.004** | **0.000** | **0.000** | 0.642 | 0.911 | 0.469 | **0.000** | **0.000** | **0.000** | **0.000** | **0.000** | **0.000** | **0.000** | **0.000** | 0.686 | **0.000** |
| **Summer** | **1-2** | 0.106 | 0.206 | 0.141 | 1.000 | 0.546 | 0.304 | 0.108 | 0.233 | 0.140 | 0.573 | 1.000 | 1.000 | 1.000 | 1.000 | 1.000 | 0.554 | 0.304 |
|  | **1-3** | 0.382 | 0.475 | 0.336 | 0.373 | 0.558 | **0.069** | 0.371 | 0.533 | 0.372 | 0.864 | 0.440 | 0.307 | 0.372 | 0.372 | 0.858 | 0.573 | **0.070** |
|  | **1-4** | **0.011** | **0.025** | **0.004** | 0.190 | 0.276 | **0.012** | **0.010** | **0.039** | **0.005** | 0.184 | 0.307 | 0.174 | 0.195 | 0.189 | 0.538 | 0.281 | **0.012** |
|  | **1-5** | **0.000** | **0.000** | **0.000** | **0.042** | **0.001** | **0.000** | **0.000** | **0.000** | **0.000** | **0.000** | **0.041** | **0.022** | **0.036** | **0.041** | 0.225 | **0.001** | **0.000** |
|  | **1-6** | 0.992 | **0.004** | **0.000** | **0.000** | **0.053** | **0.000** | 0.999 | **0.000** | **0.000** | **0.000** | **0.000** | **0.000** | **0.000** | **0.000** | **0.000** | **0.059** | **0.000** |
|  | **2-3** | 0.953 | 0.985 | 0.990 | 0.357 | 1.000 | 0.973 | 0.960 | 0.981 | 0.982 | 0.986 | 0.366 | 0.285 | 0.359 | 0.356 | 0.876 | 1.000 | 0.974 |
|  | **2-4** | 0.840 | 0.830 | 0.551 | 0.177 | 0.982 | 0.528 | 0.806 | 0.878 | 0.602 | 0.924 | 0.259 | 0.162 | 0.184 | 0.176 | 0.525 | 0.982 | 0.530 |
|  | **2-5** | **0.000** | **0.002** | **0.000** | **0.038** | **0.020** | **0.000** | **0.000** | **0.005** | **0.000** | **0.006** | **0.030** | **0.019** | **0.032** | **0.038** | 0.213 | **0.020** | **0.000** |
|  | **2-6** | 0.666 | **0.000** | **0.012** | **0.000** | 0.494 | **0.000** | 0.522 | **0.000** | **0.001** | **0.000** | **0.000** | **0.000** | **0.000** | **0.000** | **0.000** | 0.518 | **0.000** |
|  | **3-4** | 0.357 | 0.447 | 0.217 | 0.981 | 0.970 | 0.884 | 0.332 | 0.501 | 0.221 | 0.612 | 0.995 | 0.988 | 0.983 | 0.981 | 0.967 | 0.968 | 0.882 |
|  | **3-5** | **0.000** | **0.000** | **0.000** | 0.531 | **0.014** | **0.001** | **0.000** | **0.001** | **0.000** | **0.001** | 0.472 | 0.439 | 0.494 | 0.531 | 0.625 | **0.014** | **0.001** |
|  | **3-6** | 0.937 | **0.000** | **0.002** | **0.000** | 0.440 | **0.000** | 0.845 | **0.000** | **0.000** | **0.000** | **0.000** | **0.000** | **0.000** | **0.000** | **0.000** | 0.459 | **0.000** |
|  | **4-5** | **0.002** | **0.058** | **0.000** | 0.895 | 0.131 | **0.042** | **0.002** | 0.103 | **0.000** | **0.094** | 0.796 | 0.813 | 0.869 | 0.895 | 0.954 | 0.132 | **0.043** |
|  | **4-6** | 0.210 | **0.000** | 0.395 | **0.000** | 0.865 | **0.000** | 0.121 | **0.000** | 0.106 | **0.000** | **0.000** | **0.000** | **0.000** | **0.000** | **0.000** | 0.880 | **0.000** |
|  | **5-6** | **0.000** | **0.000** | 0.204 | **0.000** | 0.844 | 0.132 | **0.000** | **0.000** | 0.493 | **0.000** | **0.000** | **0.000** | **0.000** | **0.000** | **0.000** | 0.831 | 0.140 |
| **Fall** | **1-2** | 0.987 | 0.998 | 0.991 | 1.000 | 1.000 | 0.983 | 0.989 | 0.998 | 0.989 | 1.000 | 1.000 | 1.000 | 1.000 | 1.000 | 0.999 | 1.000 | 0.983 |
|  | **1-3** | **0.093** | 0.778 | **0.017** | 0.997 | **0.022** | **0.002** | 0.133 | 0.840 | **0.006** | 0.804 | 0.990 | 0.987 | 0.995 | 0.997 | 1.000 | **0.023** | **0.002** |
|  | **1-4** | **0.007** | 0.710 | **0.000** | 0.999 | **0.006** | **0.000** | **0.015** | 0.814 | **0.000** | 0.616 | 0.983 | 0.988 | 0.997 | 0.999 | 1.000 | **0.006** | **0.000** |
|  | **1-5** | 0.999 | **0.000** | **0.008** | **0.000** | **0.042** | **0.000** | 0.819 | **0.000** | **0.000** | **0.000** | **0.000** | **0.000** | **0.000** | **0.000** | **0.000** | **0.049** | **0.000** |
|  | **2-3** | **0.049** | 0.772 | **0.003** | 1.000 | **0.001** | **0.000** | **0.078** | 0.847 | **0.001** | 0.668 | 0.986 | 0.987 | 0.998 | 1.000 | 1.000 | **0.002** | **0.000** |
|  | **2-4** | **0.005** | 0.745 | **0.000** | 1.000 | **0.002** | **0.000** | **0.012** | 0.852 | **0.000** | 0.547 | 0.982 | 0.992 | 0.999 | 1.000 | 1.000 | **0.002** | **0.000** |
|  | **2-5** | 0.931 | **0.000** | **0.002** | **0.000** | **0.011** | **0.000** | 0.359 | **0.000** | **0.000** | **0.000** | **0.000** | **0.000** | **0.000** | **0.000** | **0.000** | **0.013** | **0.000** |
|  | **3-4** | 0.374 | 0.989 | **0.090** | 1.000 | 0.632 | 0.199 | 0.478 | 0.996 | **0.039** | 0.959 | 0.999 | 1.000 | 1.000 | 1.000 | 1.000 | 0.633 | 0.201 |
|  | **3-5** | **0.043** | **0.000** | 0.946 | **0.000** | 1.000 | **0.000** | **0.003** | **0.000** | 0.149 | **0.000** | **0.000** | **0.000** | **0.000** | **0.000** | **0.000** | 1.000 | **0.000** |
|  | **4-5** | **0.003** | **0.000** | 0.382 | **0.000** | 0.774 | 0.111 | **0.001** | **0.000** | 0.864 | **0.000** | **0.000** | **0.000** | **0.000** | **0.000** | **0.000** | 0.753 | 0.117 |

**Table S8.** Tukey Honestly Significant Difference (HSD) post-hoc adjusted *p*-values per Sentinel-2B band for each shrub cover group pair and season (B-Blue, G-Green, R-Red, RE-Red Edge, W. Vap.-Water Vapour). Red colored values are significant *p*-values within the 95% confidence interval (CI) (adj. *p*-value<0.05) and yellow values are those that are significant within the 90% CI, but not in the 95% CI (adj. *p*-value between 0.05 and 0.1).

|  |  | Tukey HSD post-hoc adjusted p-values | | | | | | Yell | < 0.1 | | Red | < 0.05 |
| --- | --- | --- | --- | --- | --- | --- | --- | --- | --- | --- | --- | --- |
| Season | **Shrub group pairs** | **Sentinel-2B** | | | | | | | | | | |
|  |  | **B** | **G** | **R** | **RE1** | **RE2** | **RE3** | **NIR** | **RE4** | **W. Vap.** | **SWIR 1** | **SWIR 2** |
| Spring | **1-2** | 0.779 | 0.907 | 0.565 | 0.892 | 1.000 | 1.000 | 1.000 | 1.000 | 1.000 | 0.755 | 0.471 |
|  | **1-3** | 0.534 | 0.878 | 0.548 | 0.941 | 0.994 | 0.995 | 0.997 | 0.999 | 0.994 | 0.313 | **0.051** |
|  | **1-4** | **0.001** | **0.090** | **0.001** | 0.158 | 1.000 | 1.000 | 1.000 | 1.000 | 1.000 | **0.096** | **0.019** |
|  | **1-5** | **0.000** | **0.000** | **0.000** | **0.000** | 0.484 | 0.485 | 0.567 | 0.615 | 0.666 | **0.000** | **0.000** |
|  | **1-6** | **0.000** | 0.400 | **0.000** | 0.976 | **0.000** | **0.000** | **0.000** | **0.000** | **0.000** | **0.000** | **0.000** |
|  | **2-3** | 0.999 | 1.000 | 1.000 | 1.000 | 0.996 | 0.998 | 0.999 | 0.999 | 0.989 | 0.971 | 0.828 |
|  | **2-4** | **0.011** | 0.311 | **0.036** | 0.494 | 1.000 | 0.999 | 0.999 | 0.998 | 1.000 | 0.495 | 0.330 |
|  | **2-5** | **0.000** | **0.000** | **0.000** | **0.000** | 0.442 | 0.458 | 0.525 | 0.565 | 0.595 | **0.000** | **0.000** |
|  | **2-6** | **0.000** | 0.889 | **0.000** | 0.999 | **0.000** | **0.000** | **0.000** | **0.000** | **0.000** | **0.000** | **0.000** |
|  | **3-4** | **0.015** | 0.267 | **0.019** | 0.331 | 0.992 | 0.987 | 0.984 | 0.982 | 0.986 | 0.779 | 0.787 |
|  | **3-5** | **0.000** | **0.000** | **0.000** | **0.000** | 0.601 | 0.593 | 0.650 | 0.675 | 0.799 | **0.001** | **0.000** |
|  | **3-6** | **0.000** | 0.853 | **0.000** | 1.000 | **0.000** | **0.000** | **0.000** | **0.000** | **0.000** | **0.000** | **0.000** |
|  | **4-5** | **0.000** | **0.061** | **0.000** | 0.236 | 0.515 | 0.480 | 0.512 | 0.523 | 0.655 | 0.216 | **0.008** |
|  | **4-6** | **0.000** | 0.805 | **0.000** | 0.343 | **0.000** | **0.000** | **0.000** | **0.000** | **0.000** | **0.000** | **0.000** |
|  | **5-6** | 0.469 | **0.000** | **0.000** | **0.000** | **0.000** | **0.000** | **0.000** | **0.000** | **0.000** | 0.654 | **0.000** |
| Summer | **1-2** | 0.108 | 0.236 | 0.140 | 0.558 | 1.000 | 1.000 | 1.000 | 1.000 | 1.000 | 0.548 | 0.302 |
|  | **1-3** | 0.371 | 0.541 | 0.373 | 0.851 | 0.466 | 0.303 | 0.372 | 0.372 | 0.860 | 0.562 | **0.068** |
|  | **1-4** | **0.010** | **0.041** | **0.005** | 0.167 | 0.333 | 0.173 | 0.195 | 0.189 | 0.538 | 0.275 | **0.012** |
|  | **1-5** | **0.000** | **0.000** | **0.000** | **0.000** | **0.048** | **0.022** | **0.036** | **0.041** | 0.220 | **0.001** | **0.000** |
|  | **1-6** | 0.999 | **0.000** | **0.000** | **0.000** | **0.000** | **0.000** | **0.000** | **0.000** | **0.000** | **0.053** | **0.000** |
|  | **2-3** | 0.959 | 0.980 | 0.981 | 0.987 | 0.383 | 0.281 | 0.358 | 0.355 | 0.882 | 1.000 | 0.973 |
|  | **2-4** | 0.807 | 0.884 | 0.604 | 0.914 | 0.278 | 0.161 | 0.184 | 0.176 | 0.530 | 0.982 | 0.526 |
|  | **2-5** | **0.000** | **0.006** | **0.000** | **0.005** | **0.035** | **0.019** | **0.032** | **0.037** | 0.210 | **0.019** | **0.000** |
|  | **2-6** | 0.516 | **0.000** | **0.001** | **0.000** | **0.000** | **0.000** | **0.000** | **0.000** | **0.000** | 0.493 | **0.000** |
|  | **3-4** | 0.333 | 0.507 | 0.222 | 0.596 | 0.996 | 0.989 | 0.983 | 0.981 | 0.967 | 0.969 | 0.881 |
|  | **3-5** | **0.000** | **0.001** | **0.000** | **0.001** | 0.492 | 0.435 | 0.494 | 0.531 | 0.615 | **0.014** | **0.001** |
|  | **3-6** | 0.841 | **0.000** | **0.000** | **0.000** | **0.000** | **0.000** | **0.000** | **0.000** | **0.000** | 0.439 | **0.000** |
|  | **4-5** | **0.002** | 0.109 | **0.000** | **0.082** | 0.806 | 0.808 | 0.869 | 0.895 | 0.951 | 0.131 | **0.043** |
|  | **4-6** | 0.119 | **0.000** | **0.099** | **0.000** | **0.000** | **0.000** | **0.000** | **0.000** | **0.000** | 0.866 | **0.000** |
|  | **5-6** | **0.000** | **0.000** | 0.506 | **0.000** | **0.000** | **0.000** | **0.000** | **0.000** | **0.000** | 0.843 | 0.132 |
| Fall | **1-2** | 0.989 | 0.998 | 0.989 | 1.000 | 1.000 | 1.000 | 1.000 | 1.000 | 0.999 | 1.000 | 0.982 |
|  | **1-3** | 0.133 | 0.844 | **0.006** | 0.790 | 0.991 | 0.986 | 0.995 | 0.997 | 1.000 | **0.022** | **0.002** |
|  | **1-4** | **0.015** | 0.822 | **0.000** | 0.590 | 0.984 | 0.988 | 0.997 | 0.999 | 1.000 | **0.006** | **0.000** |
|  | **1-5** | 0.820 | **0.000** | **0.000** | **0.000** | **0.000** | **0.000** | **0.000** | **0.000** | **0.000** | **0.043** | **0.000** |
|  | **2-3** | **0.078** | 0.852 | **0.001** | 0.647 | 0.988 | 0.986 | 0.998 | 1.000 | 1.000 | **0.001** | **0.000** |
|  | **2-4** | **0.012** | 0.860 | **0.000** | 0.519 | 0.984 | 0.991 | 0.999 | 1.000 | 1.000 | **0.002** | **0.000** |
|  | **2-5** | 0.362 | **0.000** | **0.000** | **0.000** | **0.000** | **0.000** | **0.000** | **0.000** | **0.000** | **0.011** | **0.000** |
|  | **3-4** | 0.478 | 0.996 | **0.037** | 0.954 | 0.999 | 1.000 | 1.000 | 1.000 | 1.000 | 0.631 | 0.200 |
|  | **3-5** | **0.003** | **0.000** | 0.130 | **0.000** | **0.000** | **0.000** | **0.000** | **0.000** | **0.000** | 1.000 | **0.000** |
|  | **4-5** | **0.001** | **0.000** | 0.878 | **0.000** | **0.000** | **0.000** | **0.000** | **0.000** | **0.000** | 0.771 | **0.099** |
